# Self‐Propelled Magnetic Micromotor‐Functionalized DNA Tile System for Autonomous Capture of Circulating Tumor Cells in Clinical Diagnostics

**DOI:** 10.1002/advs.202508636

**Published:** 2025-09-08

**Authors:** Qiumei Pu, Liangqing Lu, Yanghong Zhao, Dongxia Li, Mengli Yao, Qionglin Zhou, Xinxin Xiao, Yuzhong Jia, Xuan Zhao, Xiangde Lai, Qian Chen, Yuxiang Ji, Bin Qiao, Hua Pei, Yanan Peng, Qiang Wu

**Affiliations:** ^1^ Key Laboratory of Emergency and Trauma of Ministry of Education The First Affiliated Hospital NHC Key Laboratory of Tropical Disease Control School of Tropical Medicine & The Second Affiliated Hospital Hainan Medical University Haikou 571199 China; ^2^ International Center for Aging and Cancer Hainan Academy of Medical Sciences Hainan Medical University Haikou 571199 China; ^3^ Department of Clinical Laboratory The Second Affiliated Hospital Hainan Medical University Haikou 570311 China; ^4^ Hainan Medical University‐The University of Hong Kong Joint Laboratory of Tropical Infectious Diseases Key Laboratory of Tropical Translational Medicine of Ministry of Education School of Basic Medicine and Life Sciences Hainan Medical University Haikou 571199 China; ^5^ Public Research Laboratory Hainan Medical University Haikou 571199 China

**Keywords:** circulating tumor cells, DNA self‐assembly, enzyme‐propelled micromotors, magnetic enrichment, tumor diagnostics

## Abstract

Circulating tumor cells (CTCs) carry intact tumor molecular information, making them invaluable for personalized cancer monitoring. However, conventional capture methods, relying on passive diffusion, suffer from low efficiency due to insufficient collision frequency, severely limiting clinical utility. Herein, a magnetic micromotor‐functionalized DNA‐array hunter (MMDA hunter) is developed by integrating enzyme‐propelled micromotors, magnetic nanoparticles, and nucleic acid aptamers into distinct functional partitions of a DNA tile self‐assembly structure. This design ensured independent and compatible running of autonomous propulsion, targeted recognition, and magnetic enrichment, enabling efficient capture and subsequent identification of CTCs in clinical blood samples. The autonomous motion of the MMDA hunter is powered by O_2_ bubbles generated through the dual enzymatic cascade reactions of glucose oxidase and catalase under physiological glucose conditions. Compared to static Fe_3_O_4_ arrays (without micromotors), the MMDA hunter shows more than 2‐fold improvement in capture efficiency. Meanwhile, it achieved superb precision, simple operation, rapid response, high biocompatibility, excellent stability, and superior specificity for CTC enrichment. This method provides a reliable tool for tumor diagnosis in multiple clinical application scenarios, even in primary medical care, simultaneously offering a clever solution for the bottleneck of functional‐module interference in multifunctional nanomaterials.

## Graphical abstract

The magnetic micromotor‐functionalized DNA‐array hunter (MMDA hunter), integrating autonomous propulsion and precision‐partitioned functionalities, utilizes DNA array programmability and 5'‐3' directional control to achieve spatially partitioned module arrangement. The autonomous motion of the MMDA hunter is powered by O_2_ bubbles generated through the dual enzymatic cascade reactions of glucose oxidase and catalase under physiological glucose conditions. This system ensures independent yet compatible running of autonomous propulsion, magnetic enrichment, and targeted recognition, enabling efficient capture and precise identification of circulating tumor cells (CTCs) in clinical blood samples.

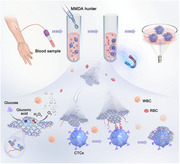

## 1 Introduction

As one of the most lethal diseases in the world, cancer faces severe challenges in clinical diagnosis and treatment due to its recurrent and metastatic characteristics.^[^ ^1^, ^2^ ^]^ While routine clinical detection methods, including magnetic resonance imaging and computed tomography, are widely utilized, their effectiveness is constrained by tumor heterogeneity and insufficient sensitivity of techniques, which makes it difficult to achieve accurate cancer diagnosis.^[^ ^3^, ^4^, ^5^ ^]^ Circulating tumor cells (CTCs), which are intact tumor cells shed into the bloodstream from primary or metastatic lesions, have significant potential as tumor markers for individualized monitoring since they carry real‐time molecular information about the tumor, and their detection not only reflects the metastatic potential of the tumor, but also provides chances to decipher genomic information through downstream analysis such as single‐cell sequencing.^[^ ^6^, ^7^, ^8^ ^]^ In the current CTCs capture technology, chemical affinity‐based approaches, like antibody or aptamers, demonstrate more improved sensitivity, accuracy, and specificity than physical ones. However, the forcible separation of antigen‐antibody complex may destroy cellular membrane integrity, thereby hampering subsequent functional analysis.^[^ ^9^, ^10^ ^]^ Nucleic acid aptamer‐based capture strategies overcome this limitation, that the biodegradable nature of nucleic acids enables easy isolation of intact cells under mild conditions. Nevertheless, current aptamer modification platforms predominantly rely on passive diffusion mechanisms, where ligand‐receptor combination is severely constrained by low‐frequency stochastic collisions in the 3D fluids, which makes it difficult to obtain the desired capture efficiency.^[^ ^11^ ^]^ Therefore, the development of capture technologies for CTCs with active movement capability is a key challenge to improve the enrichment efficiency.

By endowing nanomaterials with the characteristic of autonomous motion, micromotor technology can effectively address the limitations that traditional detection systems rely on passive diffusion to recognize and bind targets, providing a new thinking for bioassays. Among these, enzyme‐propelled micromotors—fueled by biocompatible endogenous substrates like glucose or urea—achieve self‐propulsion in physiological environments. This capability accelerates fluid mixing within microenvironments and enhances ligand‐target contact/binding efficiency, significantly shortening detection time while increasing sensitivity.^[^ ^12^ ^]^ However, classical micromotors typically employ inorganic nanomaterial carriers that lack precise spatial partition of enzymes and recognition ligands. This limitation causes functional interference due to the conflicting spatial distribution of catalytic active sites and targeting ligand domains, hindering efficient and accurate recognition in complex biofluids.^[^ ^13^, ^14^ ^]^ Consequently, there is an urgent need to develop programmable carriers that enable subregional arrangement of both micromotors and recognition ligands to achieve efficient and precise CTCs capture.

DNA tile self‐assembly, with molecular‐level manipulability and single‐base addressability, provides a superior strategy for functional integration between micromotors and recognition ligands. Compared to organic and inorganic materials, DNA tile self‐assembly exhibits highly programmability that enable incorporation into diverse assay systems.^[^ ^15^, ^16^, ^17^ ^]^ Our previous research has revealed that DNA tile self‐assembly exhibits precise assembly capabilities, serving as effective carriers for signal amplification strategies to enhance reaction kinetics, enabling sensitive detection of diverse disease biomarkers and signal‐amplified imaging of living tumor cells.^[^ ^18^, ^19^, ^20^, ^21^, ^22^, ^23^ ^]^ Meanwhile, its high efficiency and parallel assembly characteristics allow for the construction of high‐order net nanostructures at the micron level, aligning with the micrometer‐scale size requirements for CTCs capture systems.^[^ ^24^, ^25^ ^]^ Furthermore, the inherent directionality of 5′‐3′ end confers two oppositely oriented surfaces to the nanoarray, realizing divisional spatial arrangement of micromotors and capture ligands.^[^ ^26^, ^27^, ^28^ ^]^ Therefore, DNA tile self‐assembly enables precise multifunctional partitioning that fully exert the autonomous motion characteristics of propelled micromotors while guaranteeing ligand functionality, making it an ideal platform for constructing highly sensitive and selective active capture strategies.

**Scheme 1:**
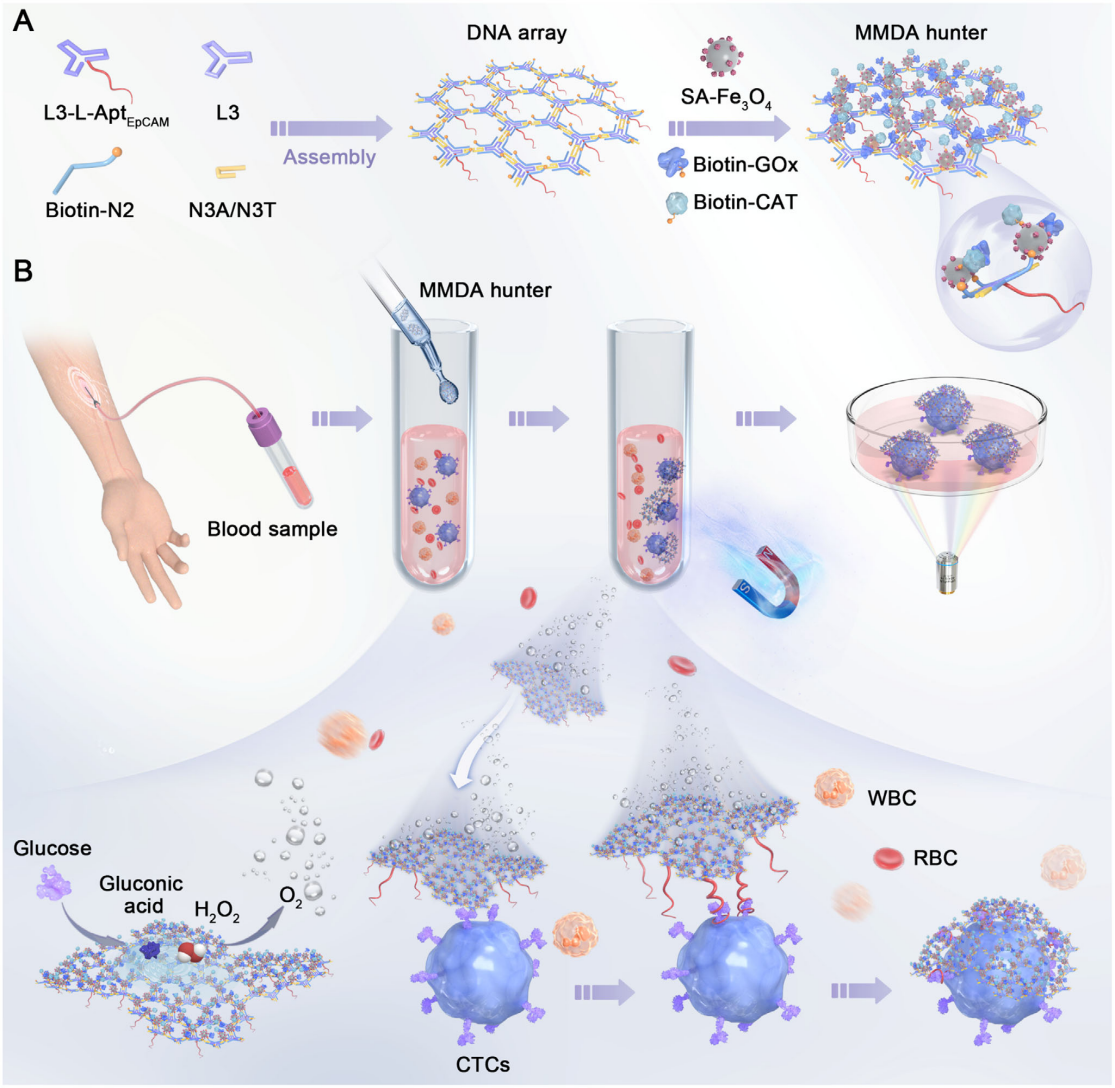
Schematic diagram of MMDA hunter assembling and capturing CTCs. A) Stepwise assembly of MMDA hunter. B) MMDA hunter captured CTCs in clinical blood samples. WBC represented white blood cell, and RBC represented red blood cell.

Herein, we constructed a magnetic micromotor‐functionalized DNA‐array hunter (named MMDA hunter) for the efficient capture and precise identification of CTCs in peripheral blood by site‐specific programming of magnetic nanoparticles, micromotors and capture ligands onto DNA arrays. As shown in **Scheme** **1**A, the structure of three‐pointed‐star DNA arrays can be constructed with only five DNA single strands, including the L3 strand, L3 with the DNA linker and EpCAM aptamer (L3‐L‐Apt_EpCAM_), biotinylated N2 strand (Biotin‐N2), and complementary paired N3 strands (N3A and N3T). Meanwhile, by taking advantage of the programmability and directionality of the 5′‐3′ ends of DNA, streptavidin‐coated Fe_3_O_4_ nanoparticles (SA‐Fe_3_O_4_), biotinylated glucose oxidase (Biotin‐GOx), and biotinylated catalase (Biotin‐CAT) were precisely immobilized on one surface of DNA arrays via streptavidin‐biotin conjugation, while the extended EpCAM aptamers were cleverly attached on the opposing surface. Fe_3_O_4_ nanoparticles provide magnetic enrichment functionality, while GOx and CAT drive motor motion through the dual enzymatic cascade reactions, and EpCAM aptamers enable targeted recognition of tumor cells. This design ensured coordinated functional operation of autonomous propulsion, magnetic enrichment, and targeted recognition among distinct partitions without mutual interference. Subsequently, peripheral blood samples from cancer patients and healthy individuals were collected to evaluate the clinical applicability of this strategy (Scheme 1B). The MMDA hunter, utilizing the dual enzymatic cascade reactions, first converted glucose to gluconic acid and H_2_O_2_ by GOx, followed by CAT‐mediated decomposition H_2_O_2_ into H_2_O and O_2_. The generated O_2_ bubbles provided the driving force for the MMDA hunter to move autonomously, significantly increasing collision frequencies between EpCAM aptamers and CTCs, ultimately enhancing targeted capture efficiency. After capture, the CTCs were rapidly enriched under magnetic field control, and then accurately identified by immunofluorescence staining. Unlike CTC capture methods relying on passive diffusion, this strategy harnesses the autonomous motion of enzyme‐propelled micromotors to overcome low collision rates between capture systems and CTCs, achieving clinical‐grade efficient, and precise CTC capture. Furthermore, the precisely organized DNA tile self‐assembly spatially segregates motion modules from detection modules, enabling both independent operation and synergistic cooperation, demonstrating the strong potential of DNA nanotechnology in medical applications.

## 2 Results and Discussion

### 2.1 Assembly and Characterization of MMDA Hunter

**Figure 1:**
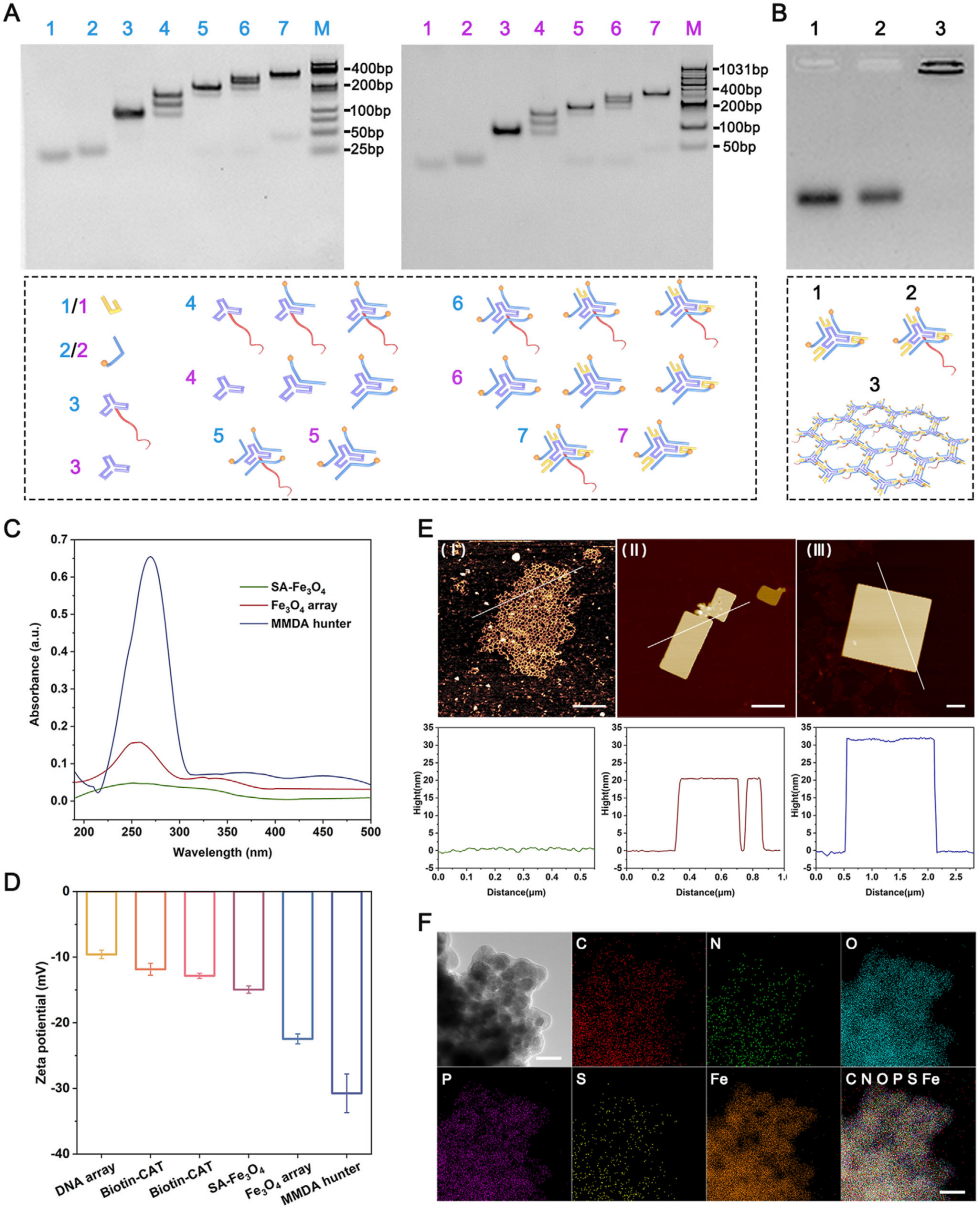
Structural characterization of MMDA hunter. A) 6% native PAGE analysis of (Left) assembly process of A tile and (Right) B tile. Lane 1, N3A/N3T; Lane 2, Biotin‐N2; Lane 3, L3‐L‐Apt_EpCAM_/L3; Lane 4, L3‐L‐Apt_EpCAM_/L3: Biotin‐N2 = 1: 1; Lane 5, L3‐L‐Apt_EpCAM_/L3: Biotin‐N2 = 1: 3; Lane 6, L3‐L‐Apt_EpCAM_/L3: Biotin‐N2: N3A/N3T = 1: 3: 1; Lane 7, L3‐L‐Apt_EpCAM_/L3: Biotin‐N2: N3A/N3T = 1: 3: 3. Schematic diagrams corresponding to the DNA bands were displayed below the 6% native PAGE images. Panel (Left): DNA marker (25–500 bp: 25, 50, 75, 100, 150, 200, 300, 400, 500 bp). Panel (Right): DNA marker (50‐1031 bp: 50, 100, 150, 200, 250, 300, 400, 500, 600, 700, 800, 900, 1031 bp). All DNA solutions were prepared at 2 µM. B) 1% AGE analysis of the assembly process of DNA arrays. Lane 1, A tile; Lane 2, B tile; Lane 3, A tile: B tile = 1: 1. Schematic diagrams of the corresponding DNA structures were shown on the right side of the 1% AGE image. All DNA solutions were prepared at 2 µm. C) UV–vis absorption spectra of SA‐Fe_3_O_4_, Fe_3_O_4_ arrays, and MMDA hunter at 190–500 nm. D) Zeta potential characterizing the assembly process of MMDA hunter. Error bars indicated the standard deviations (n = 3). E) Atomic force microscope (AFM) images and height measurements of (I) DNA arrays, (II) Fe_3_O_4_ arrays, and (III) MMDA hunter. All scar bars were 400 nm. F) TEM image of MMDA hunter and corresponding EDS elemental distribution map. All scar bars were 50 nm.

The DNA arrays, serving as the carrier for the MMDA hunter, are composed of A and B tiles. A tiles were the capture tiles carrying EpCAM aptamers targeting CTCs, while B tiles acted as the structural tiles and assembled with A tiles to construct the DNA arrays. Utilizing the orientation of the 5′‐3′ ends of DNA, biotin and extended EpCAM aptamers were modified on opposite ends of the DNA arrays. SA‐Fe_3_O_4_ was conjugated to the biotin of one surface of the hexagonal DNA array to form the Fe_3_O_4_ arrays. Biotinylated GOx and CAT are further integrated with the Fe_3_O_4_ arrays to assemble the MMDA hunter, achieving integrated functionalities of magnetic enrichment, autonomous motion, and target recognition. The successful construction of A tile, B tile, and the DNA arrays was confirmed by native polyacrylamide gel electrophoresis (PAGE) and agarose gel electrophoresis (AGE) (**Figure** **1**A,B). UV–vis absorption spectroscopy revealed characteristic absorption peaks during the stepwise assembly of the MMDA hunter. The Fe_3_O_4_ arrays exhibited a nucleic acid‐specific absorption peak at 260 nm, while MMDA hunter displayed a combined nucleic acid‐protein absorption peak at ∽270 nm^[^ ^29^, ^30^, ^31^, ^32^ ^]^ (Figure 1C). The assembly process of the MMDA hunter was monitored via zeta potential analysis (Figure 1D). All components (DNA arrays, Biotin‐GOx, Biotin‐CAT, SA‐Fe_3_O_4_) possessed inherent negative charges, resulting in progressively stronger electronegativity during the assembly process. The final zeta potential of MMDA hunter was −30.74 ± 2.95 mV, which assisted it to disperse well and stability in aqueous media.^[^ ^33^, ^34^ ^]^ Atomic force microscopy (AFM) was used to characterize the morphologies of the DNA arrays, Fe_3_O_4_ arrays, and MMDA hunter, with actual height measurements performed using NanoScope Analysis software. As shown in Figure 1E and Figure S1 (Supporting Information), the DNA arrays exhibited micron‐sized hexagonal lattice structures, while the Fe_3_O_4_ arrays formed solid sheet‐like structures due to the complete filling of the DNA nano‐lattice by Fe_3_O_4_ particles. Subsequent loading of Biotin‐GOx and Biotin‐CAT onto the Fe_3_O_4_ arrays did not significantly alter the sheet morphology but increased their height. In terms of structural thickness, the actual heights of the DNA arrays, Fe_3_O_4_ arrays, and MMDA hunter were 0.50 ± 0.21 , 20.52 ± 0.10, and 31.60 ± 0.31 nm, respectively, consistent with the heights of individual components (DNA, SA‐Fe_3_O_4_, Biotin‐GOx, and Biotin‐CAT).^[^ ^35^, ^36^, ^37^ ^]^ Confocal laser scanning microscopy (CLSM) imaging showed that green‐fluorescent‐labeled DNA arrays, red‐fluorescent‐labeled Biotin‐GOx, and orange‐fluorescent‐labeled Biotin‐CAT exhibited high co‐localization with dark, cluster‐like SA‐Fe_3_O_4_. This provides direct visualization evidence of successful assembly of the MMDA hunter (Figure S2, Supporting Information).

Transmission electron microscopy (TEM) revealed distinct morphological differences between the sheet‐like MMDA hunter and particle‐like SA‐Fe_3_O_4_. Furthermore, energy dispersive spectroscopy (EDS) detected P element– a characteristic element of DNA– within the MMDA hunter, which was absent in SA‐Fe_3_O_4_ (Figure 1F; Figure S3, Supporting Information). In conclusion, all of above results jointly demonstrated that we successfully constructed a stable and accurate MMDA hunter that can be used for subsequent CTCs capture applications.

### 2.2 Motion Analysis of MMDA Hunter

**Figure 2:**
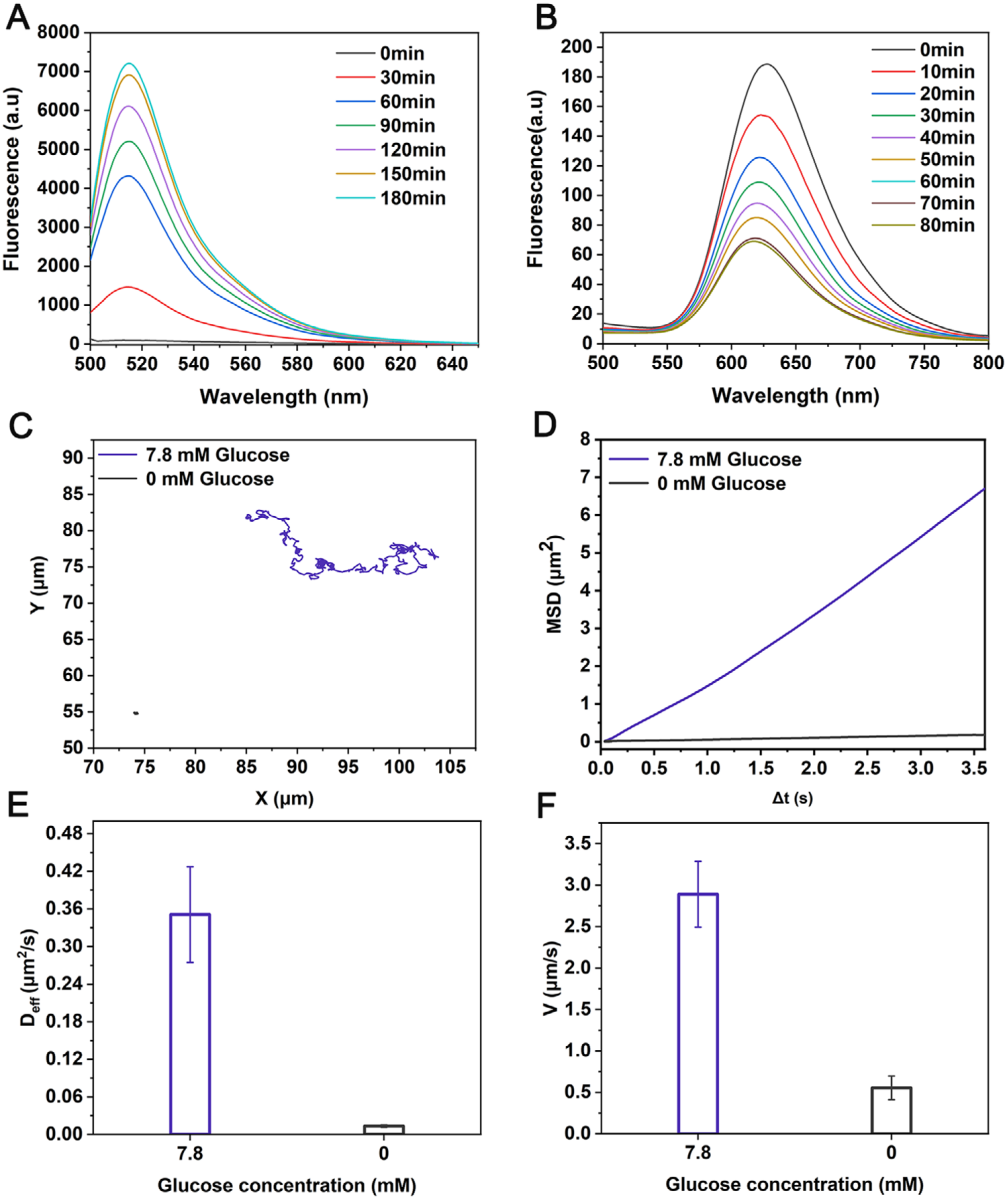
Motion analysis of MMDA hunter. A) H_2_O_2_‐sensitive fluorescent probe OxiVision™ Green verified the GOx catalytic activity of GOx‐functionalized Fe_3_O_4_ arrays. The glucose concentration was 7.80 mM and the emission wavelength was 525 nm. B) [(Ru(dpp)_3_)]Cl_2_ (fluorescence quenching O_2_ probe) detected the catalytic activity of catalase of MMDA hunter. The glucose concentration was 7.80 mM and the emission wavelength was 613 nm. C) Representative tracking trajectories of MMDA hunter in 0  and 7.80 mM glucose solutions over a period of 60 s. The optical tracking trajectories of MMDA hunter in 0  and 7.80 mM glucose solutions were obtained by optical tracking. D) Mean square displacement, E) diffusion coefficient, and F) mean velocity of the MMDA hunter in PBS/Mg^2+^ solutions without (0 mM) and with glucose (7.80 mM) were obtained by optical tracking. Error bars indicated the standard deviations (n = 5).

After the successful construction of the MMDA hunter, we first verified its chemical‐driven motion mechanism. In the catalytic system, the GOx loaded on the MMDA hunter could degrade glucose in the solution into gluconic acid and H_2_O_2_. Subsequently, through dual enzyme cascade reactions, CAT further decomposed H_2_O_2_ into H_2_O and O_2_. To evaluate the catalytic activities of these two enzymes, we used an H_2_O_2_‐sensitive fluorescent probe (OxiVision Green) and an oxygen‐indicating probe [(Ru(dpp)_3_)]Cl_2_ to monitor the real‐time fluctuation of H_2_O_2_ and O_2_ contents, respectively. As shown in **Figure** **2**A, when the Fe_3_O_4_ arrays functionalized with GOx was placed in a 7.80 mm glucose solution (postprandial glucose normal value), the fluorescence intensity of OxiVision Green continuously increased over time, indicating that GOx continuously catalyzed glucose to produce H_2_O_2_. The O_2_ monitoring experiment showed that after the MMDA hunter was added to the 7.80 mM glucose solution, it continuously produced O_2_ to gradually quench the fluorescence signal of [(Ru(dpp)_3_)]Cl_2_, and reached a fluorescence quenching plateau after 70 min, proving that the generation and consumption of O_2_ in the system reached a dynamic equilibrium (Figure 2B). These results together confirmed that the MMDA hunter could stably generate O_2_ bubbles in physiological glucose solution, providing a power basis for its autonomous movement.

Subsequently, the movement behavior of the MMDA hunter driven by glucose as fuel was evaluated. The automated microscope was used to record the movement videos of the MMDA hunter in PBS/Mg^2+^ solutions containing 0 mM glucose (control group) and 7.80 mM glucose (experimental group) (Videos S1 and S2, Supporting Information). As the results shown, the MMDA hunter exhibited classic Brownian motion in the solution with a relatively small movement range in the control group. In contrast, in the experimental group, the movement amplitude of the MMDA hunter significantly increased. Matlab code was used to track the movement trajectories of the MMDA hunter in the experimental and control groups, draw representative trajectories, and calculate the mean square displacement (MSD), diffusion coefficient (Deff), and average movement speed of the two groups based on the x and y coordinates of five trajectories in each group. As shown in Figure 2C, the representative trajectory plots showed that the experimental group had a significantly larger range of motion trajectories than the control group in 60 s. MSD analysis indicated that the control group showed an almost horizontal MSD line, consistent with the characteristics of Brownian motion. In contrast, the MSD curve of the experimental group showed a parabolic shape with a larger slope, indicating that glucose drove the MMDA hunter to transform from diffusive motion to ballistic motion,^[^ ^38^, ^39^ ^]^ which proved the bubble propelled autonomous movement mechanism (Figure 2D). Quantitative analysis showed that the diffusion coefficient of the experimental group (0.35 ± 0.08 µm^2^ s^−1^) was enhanced by 27‐fold compared with that of the control group (0.01 ± 0.00 µm^2^ s^−1^) (Figure 2E), and the average movement speed significantly increased from 0.55 ± 0.14  to 2.89 ± 0.40 µm s^−1^ (Figure 2F). In addition, we further confirmed that MMDA hunter decomposes glucose to produce oxygen bubbles through GOx and CAT double enzyme cascade catalysis, while Fe_3_O_4_ particles do not exhibit catalase‐like enzyme activity in this system and only perform magnetic enrichment functions (Figure S4 and Video S3, Supporting Information). These data fully demonstrate that the MMDA hunter exhibited an ideal autonomous movement pattern in a 7.80 mM glucose solution, and this active movement characteristic was expected to increase the collision frequency between the MMDA hunter and CTCs, thereby improving the capture efficiency.

### 2.3 Tumor Cells Capture by MMDA Hunter

**Figure 3:**
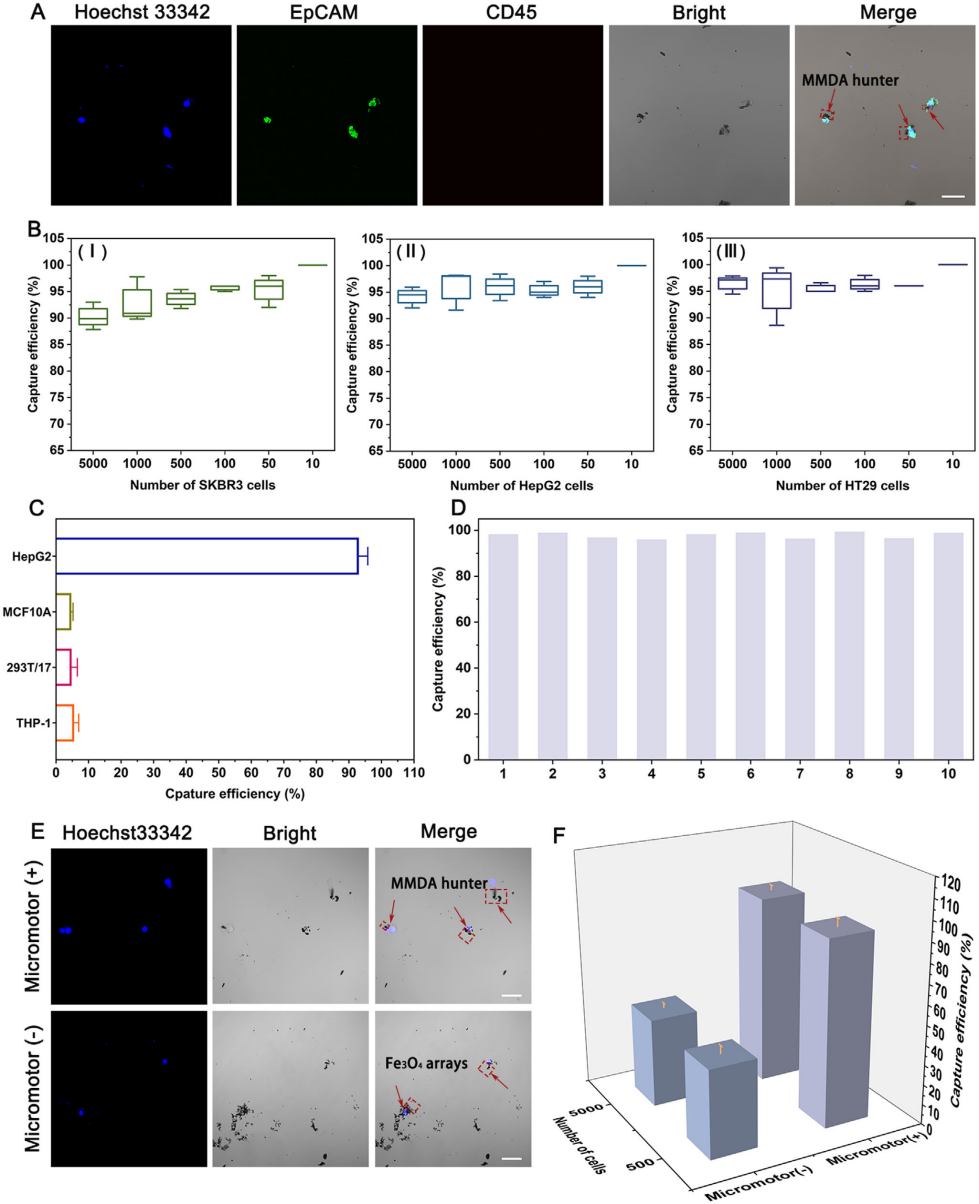
Tumor cells capture by the MMDA hunter. A) Representative CLSM images of 5000 HepG2 cells after enrichment with MMDA hunter. B) Capture efficiency of MMDA hunter for SKBR3, HepG2, and HT29 cells at different cell numbers (5000, 1000, 500, 100, 50, and 10). C) Detection specificity of MMDA hunter. Capture efficiency for 5000 HepG2, MCF10A, 293T/17, and THP‐1 cells. D) Intra‐batch repeatability of MMDA hunter. Capture efficiency of the same batch of MMDA hunter in independent experiments using 5000 HepG2 cells. E) Representative CLSM images of 5000 HepG2 cells captured by the motor system (MMDA hunter) and without‐motor system (Fe_3_O_4_ arrays). F) Comparison of capture efficiency between the motor system (MMDA hunter) and without‐motor system (Fe_3_O_4_ arrays). Capture efficiency graphs for 500 and 5000 HepG2 cells. Cells were counted using automated microscope to calculate capture efficiency. Red arrows indicated captured HepG2 cells in panels (A,E). Scar bars corresponding to 40 µm in panels A) and E). Error bars represented standard deviations (n = 3).

The viability of captured CTCs is closely related to the accuracy of downstream analysis; thus, it is crucial to ensure the high biosafety of the capture system. As shown in Figure S5 (Supporting Information), after co‐culturing Fe_3_O_4_, Fe_3_O_4_ arrays, and MMDA hunter with three types of tumor cells (SKBR3, HepG2, and HT29) for 1 h, the cell viability of all groups remained above 95.96 ± 2.24%. The MMDA hunter's extremely low cytotoxicity minimized its impacts on tumor cells, ensuring reliable subsequent analysis. The long‐term structural stability and shelf life of the detection system are critical for practical applications. UV absorption spectra of MMDA hunters from the same batch showed high overlap over 11 consecutive days, with absorbance values exhibiting only minimal increase (Figure S6, Supporting Information). This indicated negligible base exposure or protein unfolding, confirming sustained structural integrity without conformational changes. Simultaneously, we monitored DNA and enzyme concentrations of MMDA hunter under 4 °C storage from day 0 to day 10. The minimal concentration decreases observed demonstrate a shelf life of at least 11 days, meeting requirements for long‐term continuous detection (Figure S7, Supporting Information). To maximize the performance of the MMDA hunter, two parameters, working concentration and reaction time, were optimized using capture efficiency as the evaluation criterion. As shown in Figure S8 (Supporting Information), the capture efficiency was positively correlated with the concentration of MMDA hunter and reached a plateau at 100 µg mL^−1^. The optimization results of reaction time showed that the capture efficiency reached a plateau at 60 min, which was consistent with the previous data on the dynamic change of O_2_ concentration (Figure 2B). In the initial stage (0–60 min), oxygen production exceeded oxygen consumption. Hence, the MMDA hunter enhanced the active collision frequency with cells through the gradually increasing oxygen bubbles. Due to the oxygen production and consumption tended to be balanced from 60 to 80 min, the capture efficiency no longer increased and entered a stable state (Figure S9, Supporting Information). Based on this, 100 µg mL^−1^ MMDA hunter and 60 min reaction time were adopted as the optimal conditions for subsequent experiments. To visualize the capture of tumor cells by MMDA hunter, immunofluorescence staining for EpCAM (a marker for CTCs) and CD45 (a marker for white blood cells) was performed on the captured and enriched tumor cells, and light microscopy images were taken simultaneously. As shown in **Figures** **3**A and S10 (Supporting Information), it was clearly observed that the black micron‐sized MMDA hunter specifically combined to the surface of tumor cells, forming a stable composite structure. Notably, there was no obvious detachment between MMDA hunter and cells, which confirmed the effective targeting and stable binding of the interface‐modified aptamer to the EpCAM protein on the membrane surface. This characteristic could significantly reduce the risk of targeting cells loss during the detection process, thus providing a reliable guarantee for the accuracy of subsequent CTCs detection.

To comprehensively evaluate the capture ability of MMDA hunter for multiple cancers, we systematically tested its enrichment performance for three types of tumor cells from different sources (human breast cancer SKBR3, human liver cancer HepG2, and human colorectal cancer HT29) under different cell numbers. As shown in Figure 3B, for all tested cancer types, the capture efficiency remained stable above 90.56 ± 2.11%. Notably, under the condition of extremely low cell numbers (10 cells), which mimicked clinical rare CTCs, the capture efficiency of all three groups of cancer cells was as high as 100%. This result confirmed that MMDA hunter was a versatile CTC capture technology with high efficiency capture ability for various malignant tumor cells of epithelial origin. Considering the complexity of blood components, the detection specificity of MMDA hunter is crucial. Therefore, we introduced normal breast epithelial cell line MCF10A,^[^ ^40^ ^]^ human embryonic kidney cell line 293T/17,^[^ ^41^ ^]^ and monocyte cell line THP‐1^[^ ^42^ ^]^ as interfering cells.

The results showed that under the same conditions, the capture efficiency of MMDA hunter for these three types of interfering cells was 4.55 ± 0.44%, 4.78 ± 1.44%, and 5.57 ± 1.15%, while the efficiency for tumor cells HepG2 was as high as 93.03 ± 2.26% (Figure 3C). In addition, the intra‐batch repeatability experiment showed that the fluctuation range of the capture efficiency of MMDA hunter in the same batch was less than 4% in 10 independent experiments, confirming the high feasibility of this technology to stably separate rare cells from complex biological samples (Figure 3D). To clarify the contribution of autonomous movement to the capture efficiency, we compared the performance differences between MMDA hunter (with motors) and Fe_3_O_4_ arrays (without motors). Confocal laser scanning microscopy (CLSM) imaging showed that both the group with motors and the group without motors could capture HepG2 cells labeled by nuclei dye Hoechst 33342 (Figure 3E). Quantitative analysis further confirmed that regardless of whether the detection cell number was 500 or 5000, the capture efficiency of the group with motors (∽93.33 ± 3.07% or ∽95.05 ± 5.06%) was more than twice that of the group without motors (∽44.13 ± 4.20% or ∽44.57 ± 2.63%) (Figure 3F). The above results indicated that the autonomous movement strategy greatly improved the capture efficiency.

### 2.4 Detection and Analysis of CTCs in Clinical Samples Using MMDA Hunter

**Figure 4:**
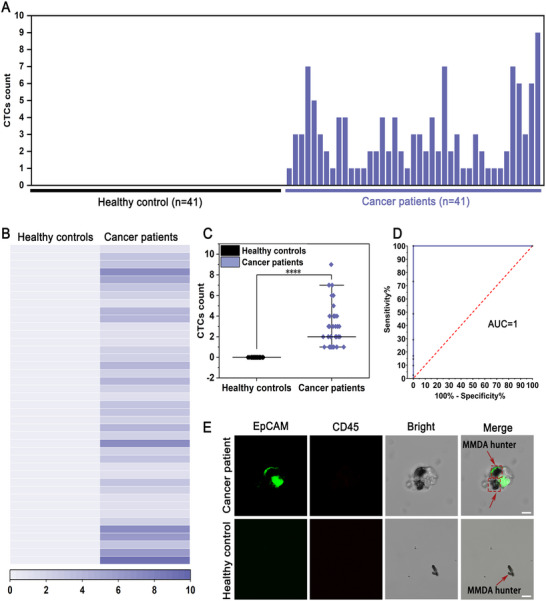
Detection of CTCs in clinical samples using MMDA hunter. A) Number of CTCs isolated from 2 mL blood samples of 41 healthy donors and 41 cancer patients. B) Heatmap analysis of the healthy control group and the cancer patient group. C) Difference in the number of CTCs between the healthy control group and the cancer patient group. The horizontal line in the middle represented the median (*P *< 0.0001). D) ROC analysis of the number of CTCs in the healthy control group and the cancer patient group. E) Representative CLSM images of CTCs isolated from blood samples of the cancer patient group and the healthy control group using MMDA hunter. The red arrows indicate MMDA hunter. Scar bars corresponding to 20 µm.

To confirm the resistance to biomolecular interference, we explored the motion of the MMDA hunter in serum. As shown in Figure S11 and Video S4 (Supporting Information), the MMDA hunter exhibited a broad range of motion, with a diffusion coefficient of 0.35 ± 0.06 µm^2^ s^−1^ and a mean velocity of 2.53 ± 0.36 µm s^−1^. These parameters are comparable to those in the buffer system, indicating that MMDA hunter maintains favorable motion performance under physiological conditions and demonstrates suitability for clinical sample analysis. In the clinical application of the MMDA hunter, we collected peripheral blood samples (2 mL per individual) from 41 patients with different cancers and 41 healthy donors. These peripheral blood samples were briefly processed,^[^ ^43^, ^44^ ^]^ and the MMDA hunter was used to capture, isolate, and count CTCs in these samples. As shown in **Figure** **4**A, no CTCs were detected in the blood samples from healthy donors, while 1–9 CTCs were isolated from the cancer patients. The heatmap results also indicated a stronger response of the MMDA hunter to the cancer patient samples (Figure 4B). Notably, the number of CTCs isolated from the cancer patient group was significantly higher than that from healthy donors (two‐sample *t*‐test; *P* < 0.0001) (Figure 4C). To demonstrate the accuracy of the assay, we plotted and calculated the area under the receiver operating characteristic curve (AUC), which was found to be as high as 1.00, indicating that this method has extremely high accuracy and reliability for cancer diagnosis (Figure 4D). The isolated CTCs were identified by immunofluorescent staining, with EpCAM and CD45 markers used to distinguish CTCs from white blood cells, effectively excluding the interference of white blood cells in CTCs counting. We observed EpCAM⁺/CD45^−^ CTCs in cancer patient samples, whereas no cells were observed under the microscope in the healthy control group only the capture system MMDA hunter was present (Figure 4E; Figure S12, Supporting Information). Benefiting from DNA degradability, captured CTCs were rapidly separated from the MMDA hunter system via simple DNase I treatment, ensuring CTCs integrity and independence for downstream analysis (Figure S13, Supporting Information). In summary, the present strategy is based on the micromotor technology, which innovatively render the capture system the ability of active movement, and the constructed MMDA hunter not only greatly improved the efficiency of CTCs capture, but also possess the advantages of simple operation, short reaction time, high biocompatibility, superb reproducibility, and excellent specificity, which can be satisfied with multi‐scenarios application mode. Therefore, we believe that MMDA hunter is expected to become a reliable technology for effective isolation and enrichment of individual CTCs in complex peripheral blood samples, providing new diagnostic tools for tumors at all stages of clinical management.

## 3 Conclusion

In this study, we designed and fabricated a micromotor‐propelled magnetic DNA nanoarray (named MMDA hunter) integrating magnetic enrichment, autonomous motility, and targeted recognition capabilities for efficient capture and precise detection of CTCs. Multi‐dimensional characterization systematically validated the assembly process and structural properties of MMDA hunter: DNA gel electrophoresis confirmed successful assembly from DNA tiles to arrays. UV–vis absorption spectroscopy and zeta potential analysis revealed the stepwise assembly process from individual components to Fe_3_O_4_ arrays and finally to MMDA hunter. AFM and EDS further confirmed its specific morphology and structural integrity, laying a foundation for subsequent functional validation. Motion mechanism studies demonstrated that increasing fluorescence intensity of the H_2_O_2_‐sensitive probe OxiVision Green and gradual quenching of the oxygen indicator [(Ru(dpp)_3_)]Cl_2_ jointly confirmed MMDA hunter's ability to generate O_2_ bubbles via the dual enzymatic cascade reactions for autonomous propulsion. Trajectory analysis and further mean square displacement, diffusion coefficient, and mean velocity analysis revealed that the MMDA hunter showed an autonomous movement pattern at physiological glucose concentration, which was conducive to enhancing the probability of collision between the MMDA hunter and the tumour cells and thus improving the capture efficiency. The biocompatibility of the MMDA hunter was verified by the CCK8 experiments, and the ultra‐low cytotoxicity of the MMDA hunter ensures the cell viability of the enriched CTCs and the accuracy of the downstream analysis results. Cellular immunofluorescence staining and light microscopy visualized MMDA hunter‐tumor cells binding, with stable complex formation. This specific binding minimized off‐target interactions during detection, ensured the accuracy of the results. After optimizing reaction conditions, MMDA hunter displayed high capture efficiency across multiple epithelial tumor cell types, confirming its potential as a universal CTCs capture platform. The use of normal cells of different origins as interference cells demonstrated the good specificity of the MMDA hunter for tumor cells, while intra‐batch reproducibility experiments showed the excellent assay stability of the MMDA hunter. Compared to the Fe_3_O_4_ arrays lacking micromotors, the active motion mechanism of MMDA hunter resulted in a substantial increase in capture efficiency. In clinical validation, MMDA hunter effectively distinguished peripheral blood samples from cancer patients and healthy donors, the active motion mechanism of MMDA hunter resulted in a substantial increase in capture efficiency. Captured CTCs were non‐destructively released via DNase I‐mediated degradation, maintaining cellular integrity for downstream analysis. Compared with other reported CTCs detection methods, this approach offers superior sensitivity due to the integration of DNA tile self‐assembly and enzyme‐propelled micromotor technology (Table S1, Supporting Information). In summary, benefiting from the autonomous motion driven by micromotors, the MMDA hunter overcomes the limitations of previous capture systems that only rely on a small range of passive diffusion to contact and target cells, and cleverly exploits the large size and programmability of the DNA tile net structure, which not only increases the capture domain, but also effectively separates different functional areas, ultimately realizing a significant increase in the detection sensitivity of CTCs within a short period of time. Meanwhile, this method, combining functionalized DNA nanotechnology with the motion characteristic of micromotors, overcomes the limitation lacking automatic targeting ability for conventional detection means, and holds the merits, like rapid, convenient and low‐cost, fully satisfy multiple clinical scenarios to provide a new idea for the innovation of clinical diagnostic of CTCs.

## 4 Experimental Section

### Materials

Oligonucleotides were synthesized from Sangon (Shanghai, CN). The DNA sequences were listed in Table S2 (Supporting Information). SA‐Fe_3_O_4_ with an average diameter of 20 nm were purchased from Zhongkekeyou (Beijing, CN). 4% (w/w) paraformaldehyde, and Cell Counting Kit‐8 were purchased from Beyotime (Shanghai, CN). The 1×TAE buffer, Hoechst 33342, Improved Minimum Essential Medium (IMEM), McCoy's 5A Medium, RPMI‐1640 Medium, DMEM Medium, fetal bovine serum (FBS), PBS buffer, 0.25% Trypsin‐EDTA, and CD45 Monoclonal Antibody (Alexa Fluor 647) were purchased from ThermoFisher Scientific (Massachusetts, USA). Biotinylated glucose oxidase, biotinylated catalase, and Tris(4,7‐diphenyl‐1,10‐phenanthroline) ruthenium(II) dichloride ([(Ru(dpp)_3_)] Cl_2_) were purchased from Med Chem Express (New Jersey, UA). EpCAM Rabbit Monoclonal Antibody (Alexa Fluor 488) was purchased from Abcam (Cambridge, UK). Human cell lines SKBR3 (RRID: CVCL_0033), HT29 (RRID: CVCL_0320), HepG2 (RRID: CVCL_0027), MCF10A (RRID: CVCL_0598), THP‐1 (RRID: CVCL_0006), and 293T/17 (RRID: CVCL_1926) were purchased from Procell (Wuhan, CN). All cell lines were contamination free. OxiVision Green hydrogen peroxide sensor was purchased from AAT Bioques (California, US).

### Assembly of MMDA Hunter

Relevant ratios of 2 µM DNA single strands in 1×TAE/Mg^2+^ buffer (40 mM Tris base, 20 mM acetic acid, 2 mM EDTA, and 12.5 mM MgCl_2_; pH 8.0) were assembled into the DNA tiles via heating at 95 °C for 5 min and slowly annealing to 10 °C. The DNA tiles A and B were mixed in a 1: 1 ratio and annealed from 33 to 10 °C, forming the DNA array. The SA‐Fe_3_O_4_ (100 µg mL^−1^) were connected to the DNA array (2 µM) in the coupling buffer (10 mM Tris‐HCl, 12.5 mM MgCl_2_, 1 M NaCl, 1 mM EDTA, and 0.05% Tween‐20 (v/v); pH 7.4), and then biotinylated glucose oxidase (1000 µg mL^−1^) and biotinylated catalase (334 µg mL^−1^) were added to construct the MMDA hunter. All the reactions were carried out under mild shaking conditions at room temperature.

### Motion Experiments

The MMDA hunter (100 µg mL^−1^) was added to 100 µL of 7.80 mM glucose solution and PBS/Mg^2+^ buffer (12.5 mM MgCl_2_; pH 7.4) respectively and added to 96‐well plate. The movement of MMDA hunter was observed and recorded using the automated microscope (Lionheart FX, Agilent, USA) with a bright field of view at 37 °C. The recorded videos were imported into Matlab (v. R2023a, Mathworks, Inc., USA) where the particle trajectories, velocity of movement, mean square displacement, and diffusion coefficient were analyzed according to the protocol published elsewhere.

### The Capture for Tumor Cells Using MMDA Hunter

Different numbers of cells (5 × 10^3^, 1 × 10^3^, 5 × 10^2^, 1 × 10^2^, 5 × 10, 1 × 10 cells) were spiked into the concentration of 7.80 mM glucose solution, and then the MMDA hunter was added to incubate for 1 h at 37 °C. The captured cells were separated and collected using a magnetic separation rack. After removal of the supernatant and washing three times, the captured cells were resuspended in PBS/Mg^2+^ buffer (pH 7.4). The spiked cell suspensions and captured cell suspensions were added to 48‐well plates, and the cells were counted using an automated microscope (Lionheart FX, Agilent, USA). The capture efficiency was defined as the ratio of captured cells against the total number of spiked cells.

### The Capture of CTCs in Clinical Peripheral Blood Samples Using MMDA Hunter

Patients who were diagnosed with cancer were taken into the experimental group, and the healthy people were integrated into the control group at the same period. Relevant information of all clinical peripheral blood samples was shown in Tables S3 and S4 (Supporting Information). The ethylenediaminetetraacetic acid (EDTA) anticoagulated bloods (2 mL) were briefly treated following the methods described in previous articles.^[^ ^43^, ^44^ ^]^ Subsequently, CTCs were captured as previously described above. The captured CTCs were identified with immunofluorescence as previously described. Only the cells with phenotypes of EpCAM positive but CD45 negative were identified as CTCs.

### Ethical Statement

The peripheral blood samples from healthy persons and cancer patients being used in the study were from the Second Affiliated Hospital, Hainan Medical University. The Ethics Committee of Hainan Medical University approved all experimental process of this study, and waived the requirement for informed consent (Approval Number: HYLL‐2022‐224).

### Statistical Analysis

All experimental data were recorded from at least three independently repeated samples and denoted as mean ± standard deviation (SD). Data analysis was performed using Origin Pro 2024 software (version 10.1.0). Statistical comparisons between two samples were performed using a two‐sample *t*‐test. A significant statistical difference between two groups was considered when *P* < 0.05. The p‐values corresponded to ^****^ *p* < 0.0001, ^***^ *p* < 0.001, ^**^ *p* < 0.01, ^*^ *p* < 0.05, and ns: *P* > 0.05.

Other methods are described in the Supporting Information.

## Conflict of Interest

The authors declare no conflict of interest.

## Supporting information



Supporting Information

Supplemental Video 1

Supplemental Video 2

Supplemental Video 3

Supplemental Video 4

## Data Availability

The data that support the findings of this study are available from the corresponding author upon reasonable request.
