# Supplementary material for: Self‐Propelled Magnetic Micromotor‐Functionalized DNA Tile System for Autonomous Capture of Circulating Tumor Cells in Clinical Diagnostics
Source: Adv Sci (Weinh). 2025 Sep 8;12(45):e08636. doi: 10.1002/advs.202508636 (PMC12677621; doi:10.1002/advs.202508636)
Supplement: Supplementary file 1 — Supporting Information [file ADVS-12-e08636-s001.docx]

Supporting Information

Self-Propelled Magnetic Micromotor-Functionalized DNA Tile System for Autonomous Capture of Circulating Tumor Cells in Clinical Diagnostics

Qiumei Pu, Liangqing Lu, Yanghong Zhao, Dongxia Li, Mengli Yao, Qionglin Zhou, Xinxin Xiao, Yuzhong Jia, Xuan Zhao, Xiangde Lai, Qian Chen, Yuxiang Ji, Bin Qiao, Hua Pei*, Yanan Peng*, and Qiang Wu*

Q. Pu, L. Lu, Y. Zhao, X. Zhao, X. Lai, Q. Chen, Y. Ji, B. Qiao, Q. Wu

Key Laboratory of Emergency and Trauma of Ministry of Education, The First Affiliated Hospital; NHC Key Laboratory of Tropical Disease Control, School of Tropical Medicine & The Second Affiliated Hospital, Hainan Medical University, Haikou 571199, China

E-mail: wuqiang@muhn.edu.cn

D. Li, Y. Peng

International Center for Aging and Cancer, Hainan Academy of Medical Sciences, Hainan Medical University, Haikou 571199, China

E-mail: pengyanan2022@muhn.edu.cn

M. Yao, H. Pei

Department of Clinical Laboratory, The Second Affiliated Hospital, Hainan Medical University, Haikou 570311, China

E-mail: phzmh61@aliyun.com

Q. Zhou, X. Xiao

Hainan Medical University-The University of Hong Kong Joint Laboratory of Tropical Infectious Diseases, Key Laboratory of Tropical Translational Medicine of Ministry of Education, School of Basic Medicine and Life Sciences, Hainan Medical University, Haikou 571199, China

Y. Jia

Public Research Laboratory, Hainan Medical University, Haikou 571199, China

Experimental Section

**Methods**

**1.1. Electrophoresis Analysis**

The 6% and 12% native PAGE were conducted using vertical electrophoresis systems (Bio-Rad, USA), and 1% AGE was conducted using horizontal electrophoresis systems (Tanon, China). The 1×TAE/Mg^2+^buffer was the running buffer, and gels were run at 4°C for1-2 h at a constant pressure of 90-110 V. The gels were stained with Super Red Dye for 40 min, following the image via the Gel imaging analysis system (ABLX5, Tanon, China).

**1.****2.** **UV Absorbance Spectra**

Fe_3_O_4_, Fe_3_O_4_ array and MMDA hunter were respectively diluted with coupling buffer, yielding a final concentration of Fe_3_O_4_ of 100 μg/mL in each group. The absorbance spectra were recorded in the absorbance mode over the wavelength range of 190-500 nm. A blank solution of coupling buffer was used as a reference, and the background absorbance was subtracted from the sample absorbance. The results were obtained using ultraviolet-visible (UV-vis) spectrophotometer (T6, Beijing Purkinje GENERAL Instrument Co., Ltd, CN).

**1.3. Zeta Potential Measurements**

The zeta potential of the Fe_3_O_4_, biotinylated glucose oxidase, biotinylated catalase, DNA array_,_ Fe_3_O_4_ arrays, and MMDA hunter were measured using multi-angle particle size and Zeta potential analyzer (Omni, Brookhaven Instruments, USA). The samples were prepared by diluting the original suspensions with coupling buffer. Before measurement, the samples were gently stirred and then transferred into a disposable cuvette, ensuring no air bubbles were introduced, as air bubbles could interfere with the measurement. The temperature during the measurement was maintained at 25.00 ± 0.10°C.

**1.4. Atomic Force Microscopy**

The DNA array (1 μM, 10 μL), Fe_3_O_4_ array (100 μg/mL, 10 μL), and MMDA hunter (100 μg/mL, 10 μL) were each diluted 20 folds, subsequently deposited onto the surface of freshly cleaved mica, incubated for 2-3 minutes, rinsed with ultrapure water for 2-3 times, and air-dried at room temperature. Atomic Force Microscopy (AFM) images were obtained using bruker dimension icon (Bruker company, USA) with SCANASYST-AIR Probe (Bruker company, USA). The AFM images were analyzed by NanoScope Analysis software (Version 3.0, Bruker company, USA).

**1.5. Confocal Laser Scanning Microscopy Characterization for MMDA Hunter**

DNA arrays, Biotin-GOx, and Biotin-CAT were labeled with Alexa Fluor 488, Alexa Fluor 647, and Alexa Fluor 594, respectively. Subsequently, the MMDA hunter incorporating these three fluorescent labels was constructed, and colocalization imaging was performed using confocal laser scanning microscopy (CLSM). CLSM images were obtained by FV3000 Laser Scanning Microscopy (Olympus, Japan). The Alexa Fluor 488 fluorescence channel has a maximum excitation wavelength of 499 nm and a maximum emission wavelength of 520 nm. The Alexa Fluor 647 channel has a maximum excitation wavelength of 650 nm and a maximum emission wavelength of 668 nm. The Alexa Fluor 594 channel has a maximum excitation wavelength of 590 nm and a maximum emission wavelength of 617 nm.

**1.6. Transmission Electron Microscopy and Energy Dispersive Spectroscopy**

The MMDA hunter (100 μg/mL, 10 μL) were diluted 20 folds and added on a copper grid, stained with 1% phosphotungstic acid solution. The operating conditions included an accelerating voltage of 300 kV and a magnification range from 10,000× to 500,000×. The characterization images were obtained using transmission electron microscopy (TEM, Tecnai G2 F30, FEI company, USA) and energy dispersive spectroscopy (EDS, Ultim Extreme, OXFORD Instruments, UK).

**1.7. Fluorescence Spectra**

OxiVision™ Green hydrogen peroxide sensors (100 μL, 10 μM) were mixed with 200 μg/mL of biotinylated glucose oxidase modified Fe_3_O_4_ array. The mixture was then added to the glucose solution in a 1:1 ratio, which gave a final concentration of 7.80 mM of glucose. The fluorescence intensities were measured at the excitation/emission wavelengths of 490/525 nm. [(Ru(dpp)_3_)] Cl_2_ nanoprobes (100 μL, 100 μg/mL) were mixed with 200 μg/mL of MMDA hunter. The mixture was added in equal ratios to a glucose solution with a final concentration of 7.80 mM. The emission at 500-800 nm was recorded after excitation at 463 nm. All emission spectrums were measured by a fluorescence spectrophotometer (F-4700, Hitachi, Japan)

**1.8. Cell Culture**

The HepG2 cells were cultured in IMEM Medium supplemented with 10% FBS and 1% penicillin/streptomycin. The HT-29 and SKBR3 cells were cultured in McCoy's 5a Medium with 10% FBS and 1% penicillin/streptomycin. The THP-1 cells were cultured in RPMI-1640 Medium with 10% FBS, 0.05 mM 2-mercaptoethanol and 1% penicillin/streptomycin. The 293T/17 cells were cultured in DMEM Medium supplemented with 10% FBS and 1% penicillin/streptomycin. The MCF10A cells were cultured in DMEM/F12 Medium with 5% HS, 20 ng/ml epidermal growth factor, 0.50 μg/ml Hydrocortisone, 10 μg/mL Insulin, 1% NEAA and 1% penicillin/streptomycin. All cells were cultured at 37°C in 5% CO_2_.

**1.9. Evaluation of Cell Viability**

The SKBR3, HepG2 and HT29 cells were seeded at 5×10^3^ cells/well in 96-well plates for 24 h, respectively. Then, the cells were incubated with three groups of Fe_3_O_4_, Fe_3_O_4_ array and MMDA hunter at a final concentration of 100 μg/mL for 1 h at 37°C. The CCK-8 solution (10 μL) was added to the cells for 4 h at 37°C. The absorbance at 450 nm was recorded via a Multimode Reader (SynergyHTX, BioTek, USA).

**1.10. Immunofluorescence Identification of the Captured Tumor Cells**

The captured cells were collected and then fixed with 4% paraformaldehyde for 20 min, permeabilized with 0.1% Triton-X 100 for 10 min, blocked with 1% BSA for 30 min, incubated with the EpCAM Monoclonal Antibody (Alexa Fluor 488) and CD45 Monoclonal Antibody (Alexa Fluor 647) for overnight at 4°C and finally stained Hoechst 33342 for 10 min. The immunofluorescence images were captured using a laser confocal scanning microscope (FV3000, Olympus Corporation, Japan).


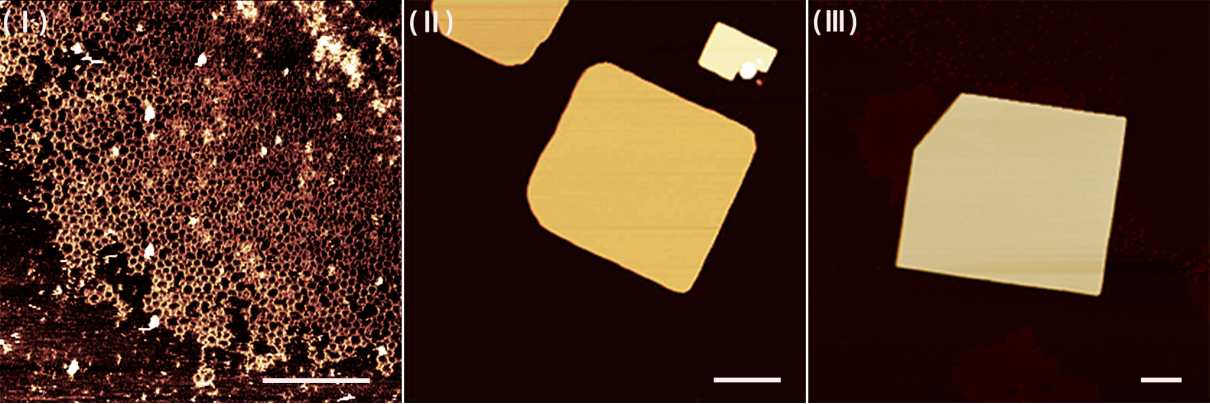


Figure S1. AFM images of (I) DNA arrays, (II) Fe_3_O_4_ arrays and (III) MMDA hunter. All scale bars were 400 nm.

**
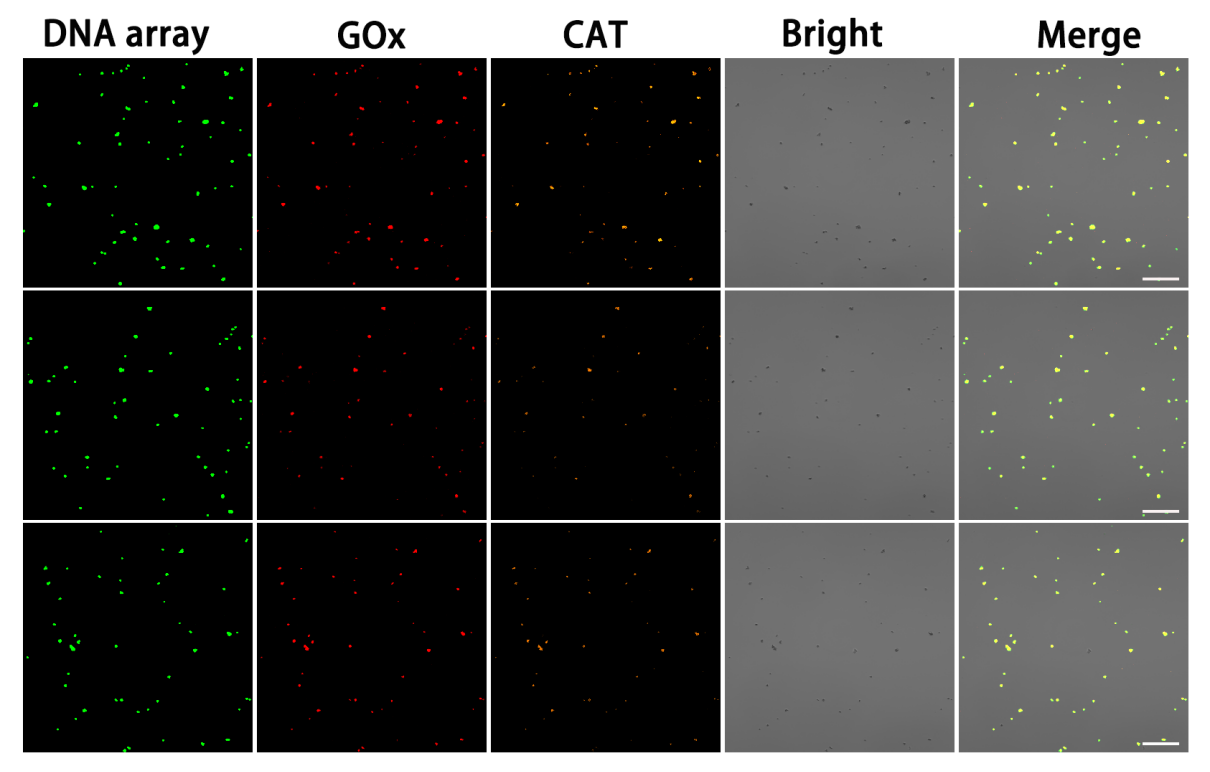
**

**Figure S2.** CLSM images of MMDA hunter (n=3). The scale bars were 50 μm.


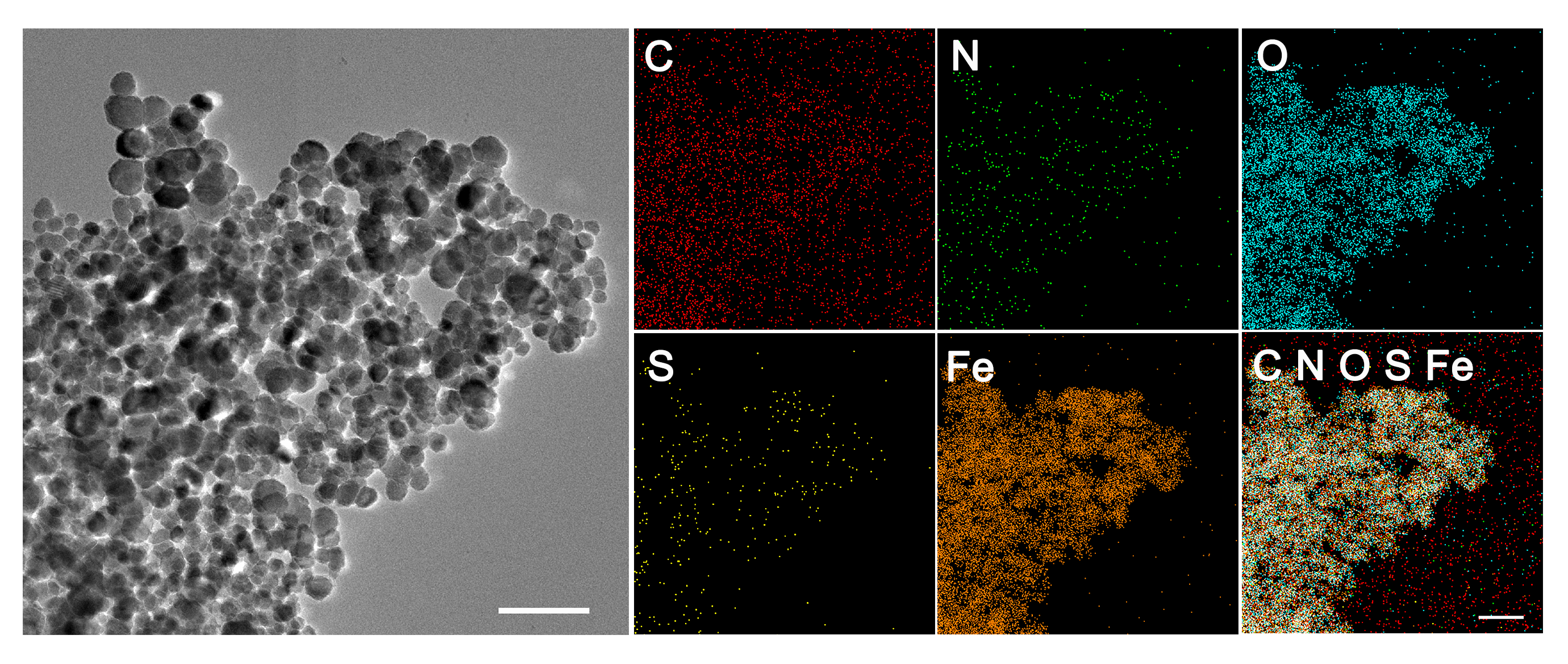


Figure S3. TEM image of SA-Fe_3_O_4_ and corresponding EDS elemental distribution map. All scale bars were 100 nm.


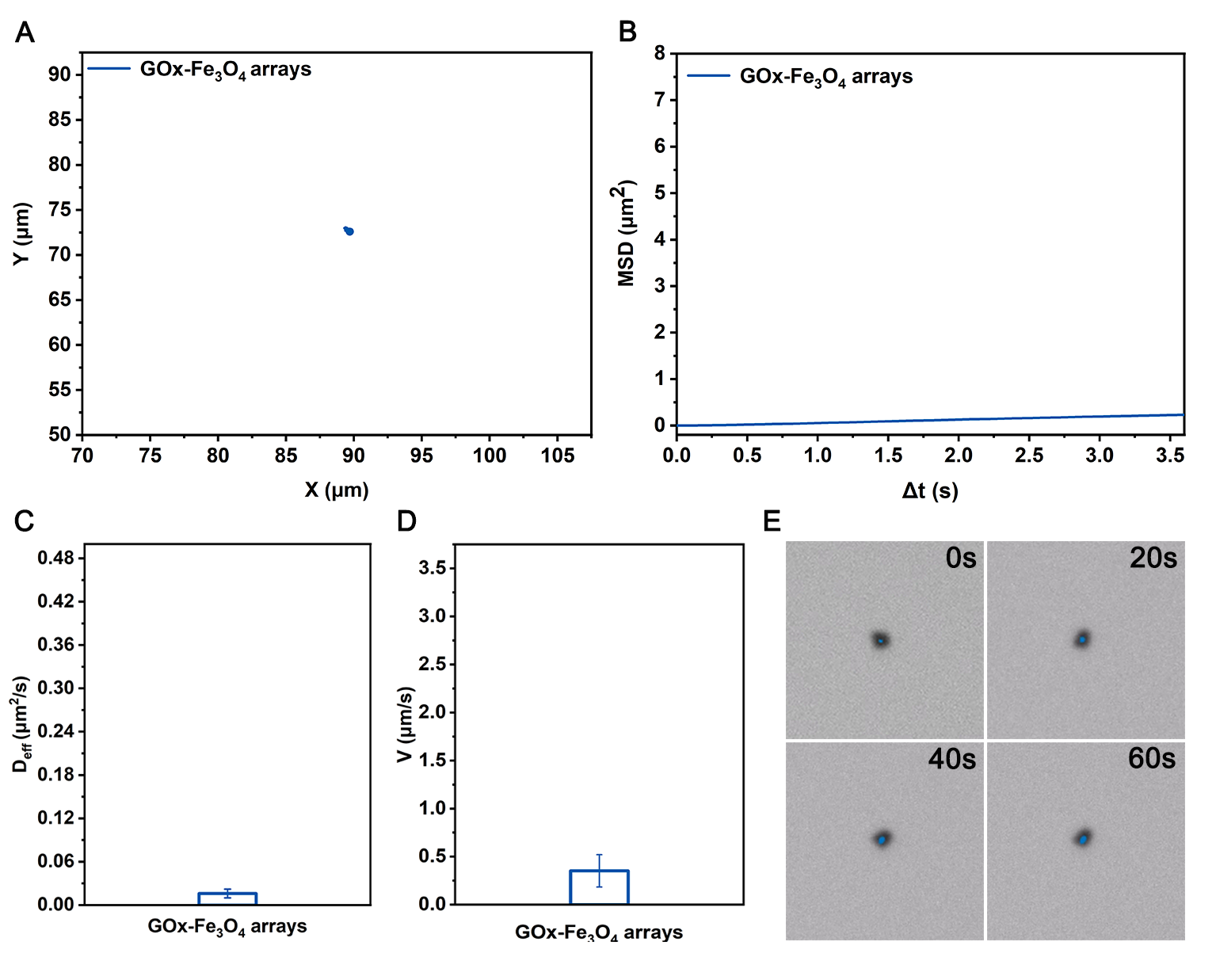


Figure S4. A) Representative tracking trajectories of GOx-Fe_3_O_4_ arrays in 7.80 mM glucose solutions over a period of 60 s. The optical tracking trajectories of GOx-Fe_3_O_4_ arrays in 7.80 mM glucose solutions were obtained by optical tracking. B) Mean square displacement, C) diffusion coefficient, and D) mean velocity of the GOx-Fe_3_O_4_ arrays in 7.80 mM glucose solutions were obtained by optical tracking. E) Representative tracking trajectory images of GOx-Fe_3_O_4_ arrays in 7.80 mM glucose solution at 0, 20, 40, and 60 s. Error bars indicated the standard deviations (n=5).


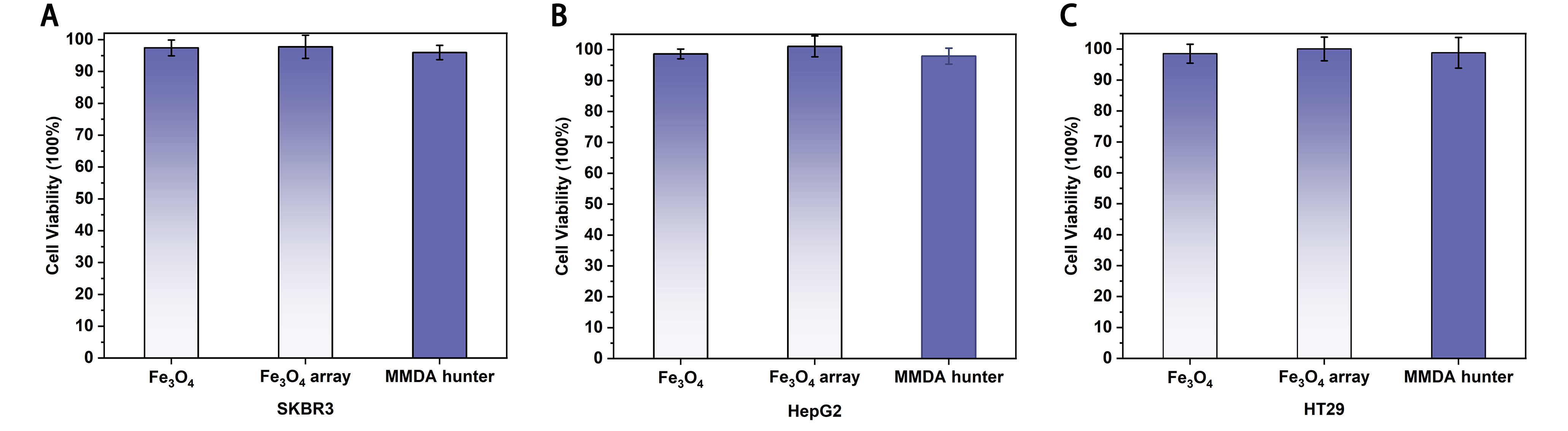


**Figure S5. Cytotoxicity evaluation of Fe_3_O_4_, Fe_3_O_4_ arrays, and MMDA hunter.** A) SKBR3, B) HepG2, and C) HT29 cells were incubated with Fe_3_O_4_ (100 μg/mL), Fe_3_O_4_ arrays, or MMDA hunter at 37℃ for 1 h. Cell viability was measured. Error bars represented standard deviations (n=3).


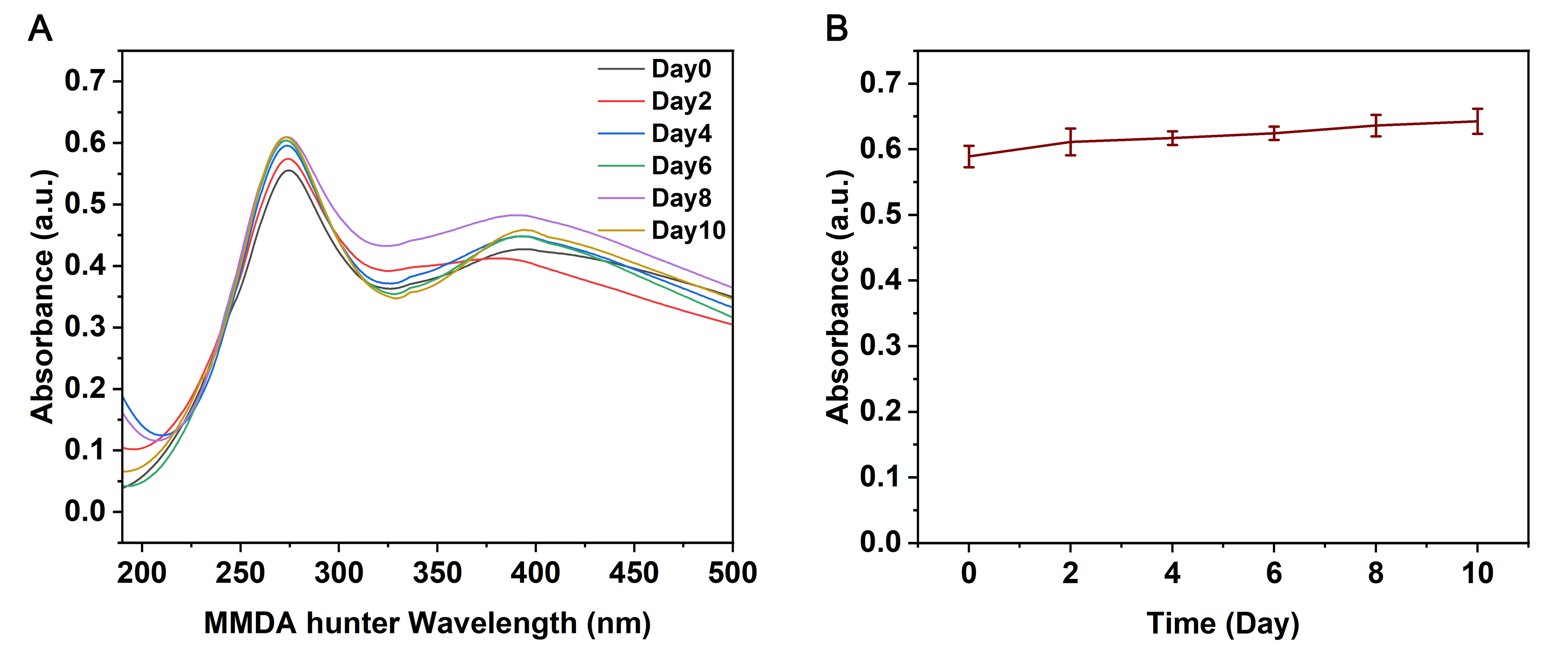


Figure S6. UV-vis spectrum of MMDA hunter. (A) Full-range spectra (190-500 nm) and (B) absorption peak values at 270 nm were monitored daily over 11 days for the same MMDA hunter batch stored at 4°C. Error bars indicated the standard deviations (n=3).


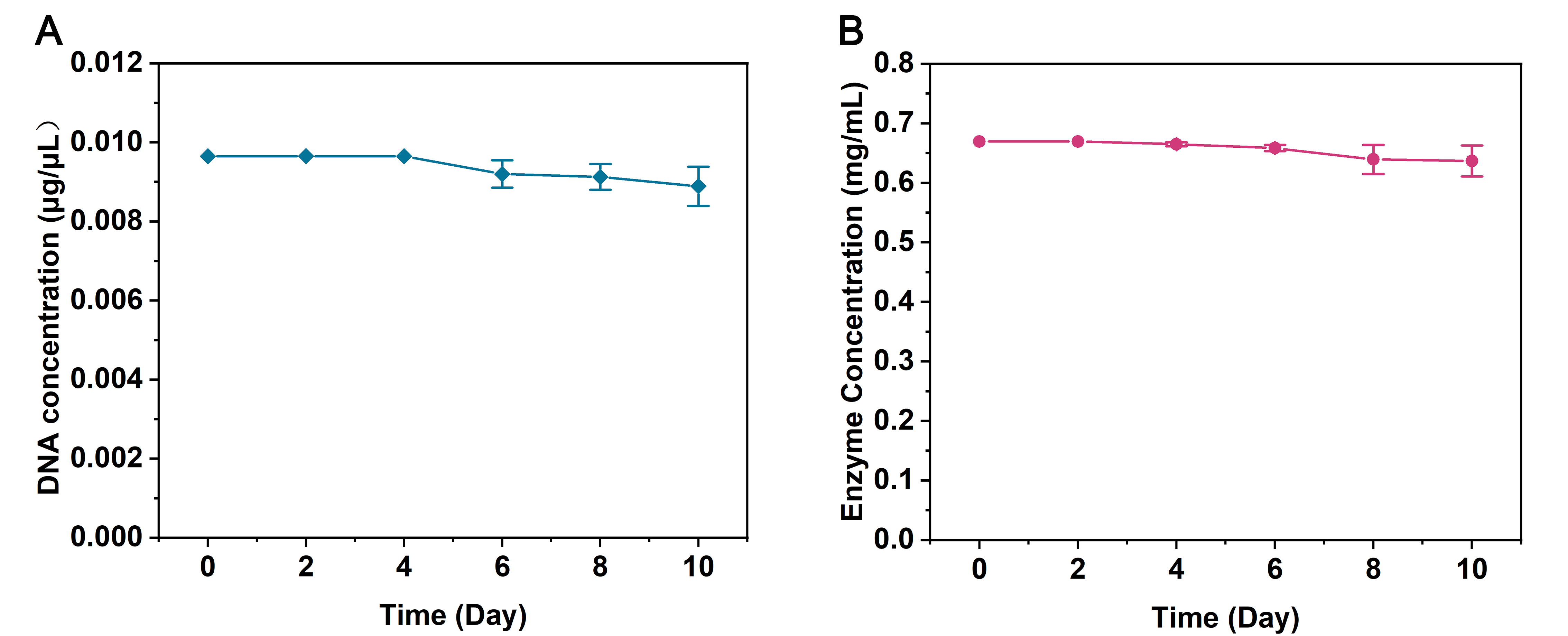


Figure S7. The same batch of MMDA hunter was stored at 4°C, and the (A) DNA concentration and (B) enzyme concentration of the same MMDA hunter sample was continuously monitored for 11 days. Error bars indicated the standard deviations (n=3).


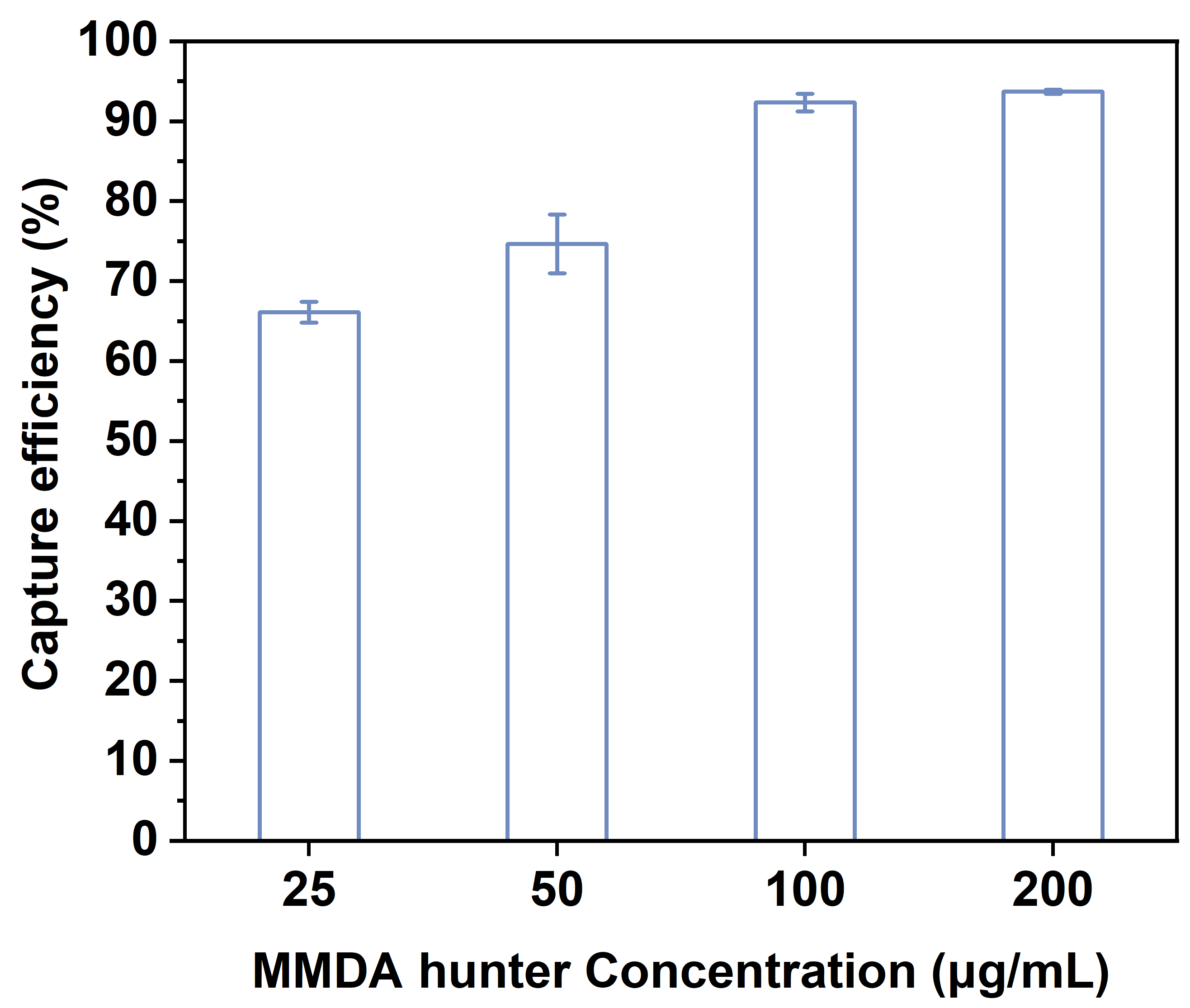


**Figure S8. Optimization of MMDA hunter working concentration.** Capture efficiency comparison of four concentrations of MMDA hunter (25, 50, 100, and 200 μg/mL) incubated with 5000 SKBR3 cells at 37°C for 1 h. Cells were counted using an automatic live-cell imaging system to calculate capture efficiency. Error bars represented standard deviations (n=3).


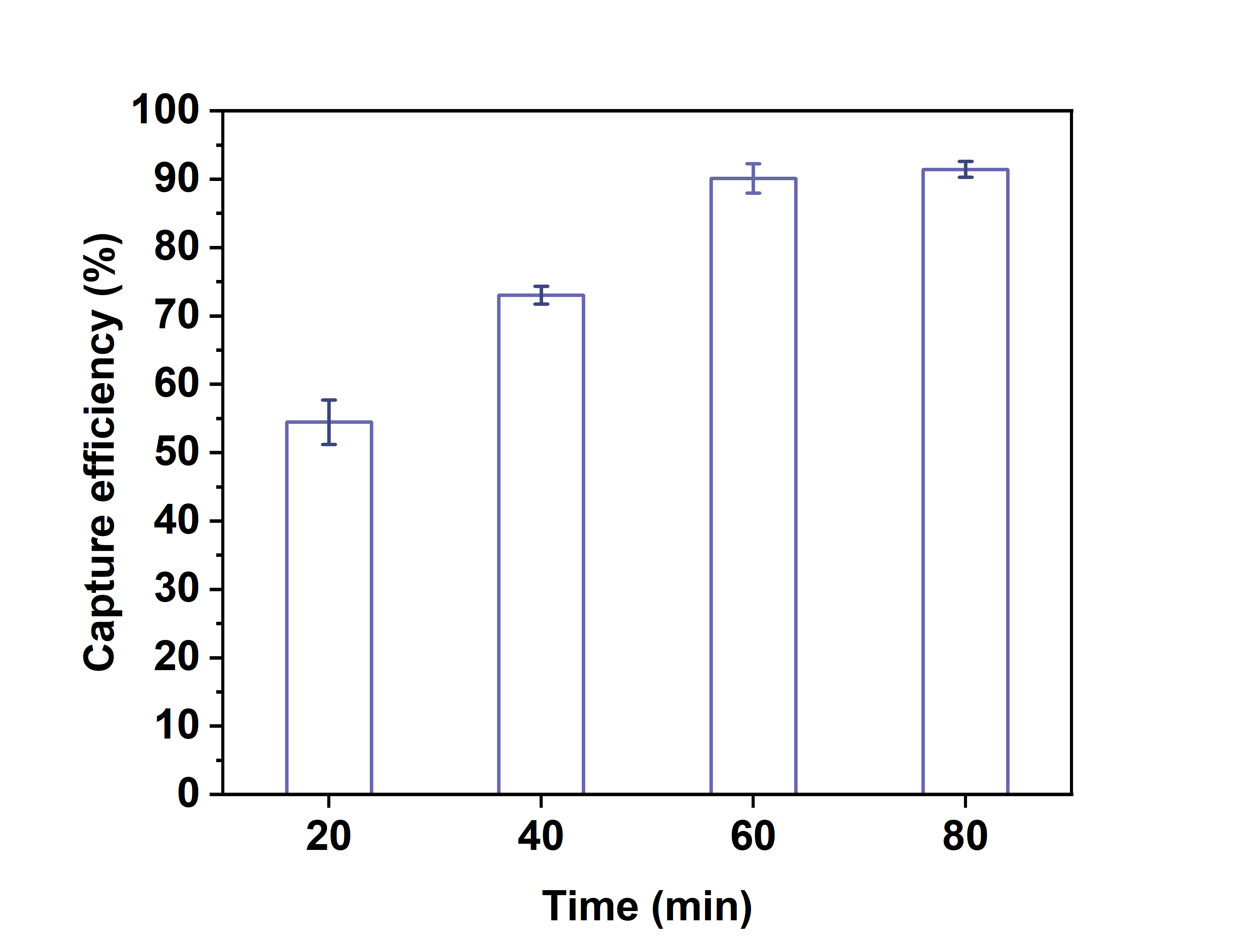


**Figure S9. Optimization of capture time for MMDA hunter.** Comparison of capture efficiency of MMDA hunter incubated with 5000 SKBR3 cells at 37℃ for different time (20, 40, 60, and 80 min). Cells were counted using an automatic live-cell imaging system to calculate capture efficiency. Error bars represented standard deviations (n=3).


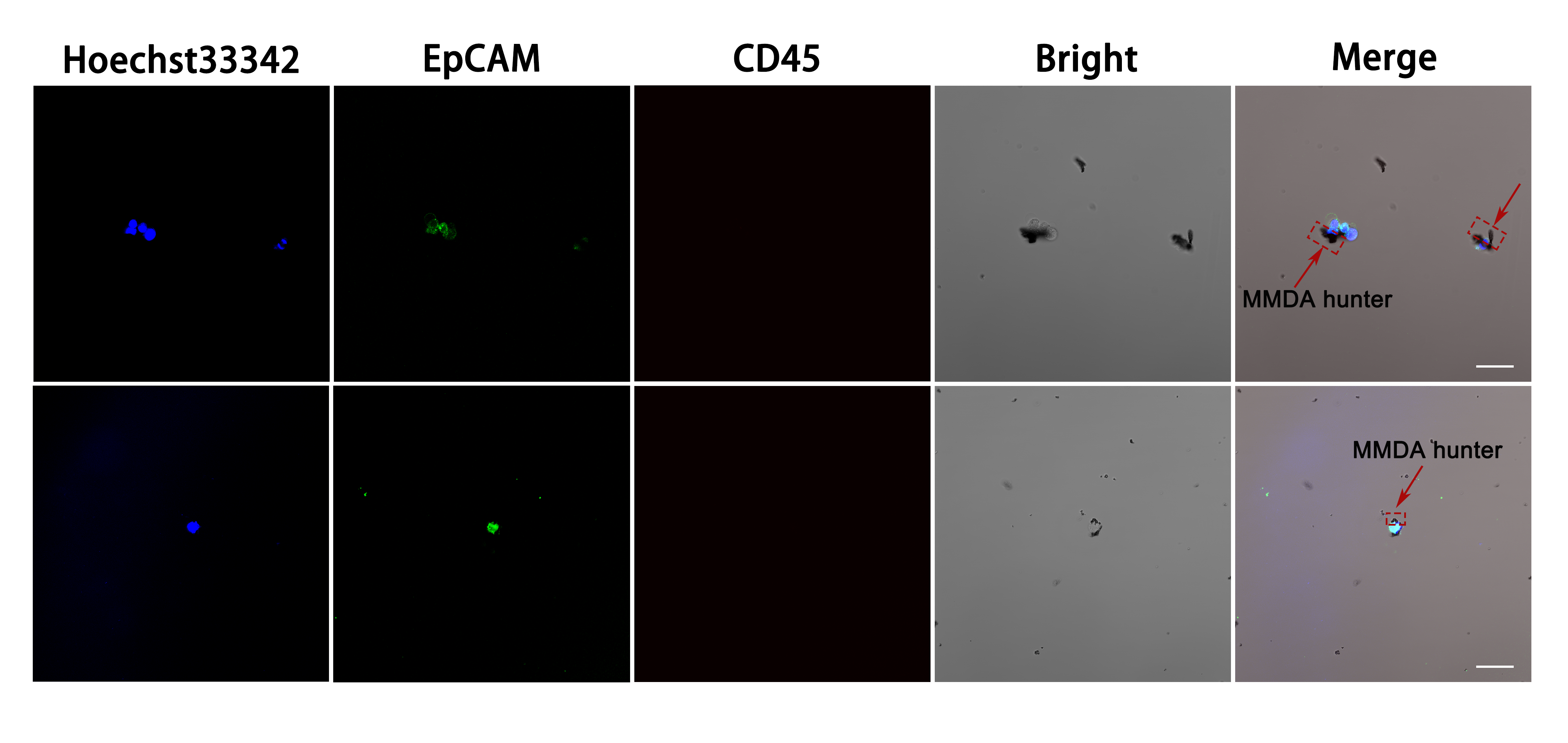


**Figure S10. Representative CLSM images of MMDA hunter capturing 5000 HepG2 cells.** All scale bars = 40 μm.


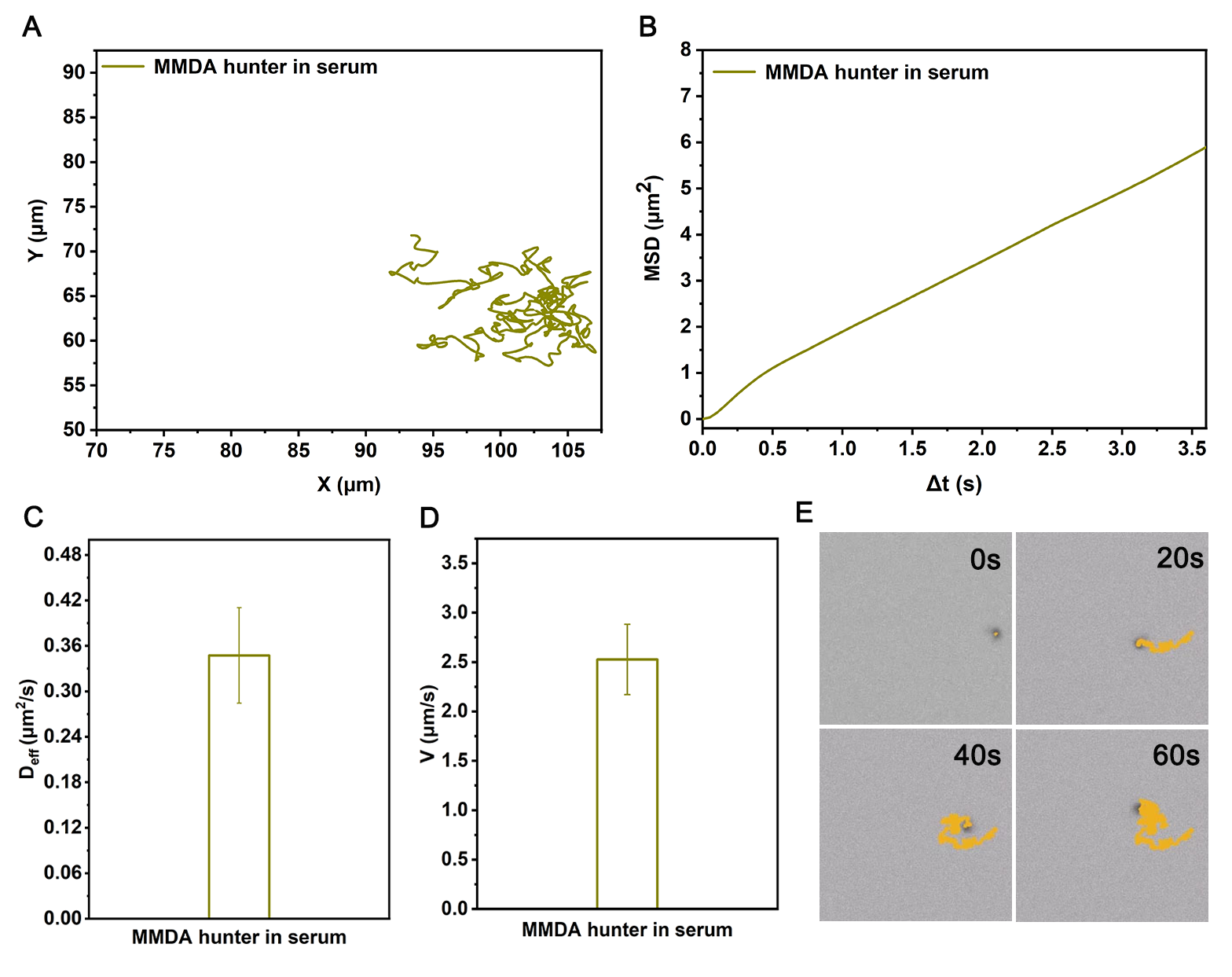


Figure S11. A) Representative tracking trajectories of MMDA hunter in serum over a period of 60 s. The optical tracking trajectories of MMDA hunter were obtained by optical tracking. B) Mean square displacement, C) diffusion coefficient, and D) mean velocity of MMDA hunter in serum were obtained by optical tracking. E) Representative tracking trajectory diagrams of MMDA hunter in serum at 0, 20, 40, and 60 s. Error bars indicated the standard deviations (n=5).


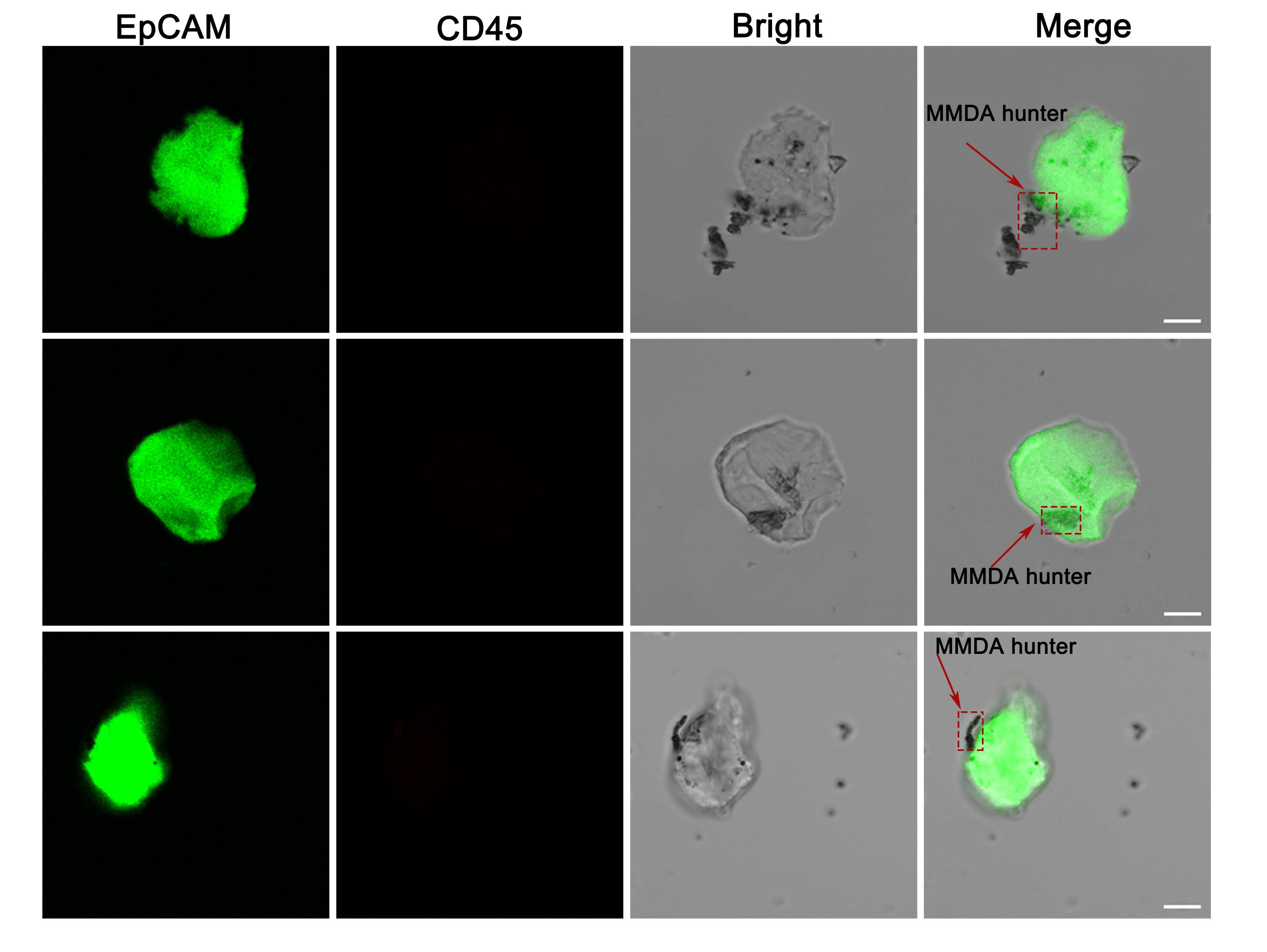


**Figure S12. Representative CLSM images of CTCs isolated from blood samples of cancer patients using MMDA hunter.** Red arrows indicated MMDA hunter. All scale bars = 10 μm.


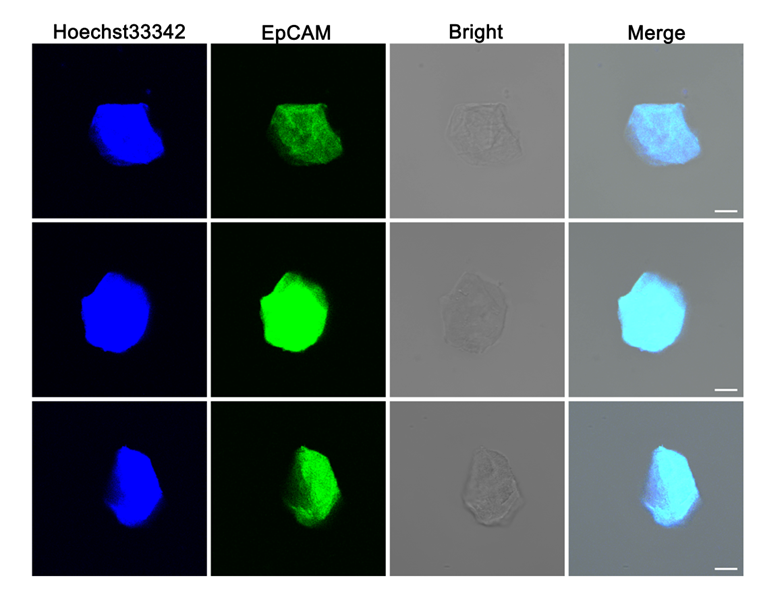


**Figure S13. Representative CLSM images of intact CTCs obtained from cancer patient blood samples using MMDA hunter followed by DNase I treatment**. All scale bars = 10 μm.

**Table S1. Comparison of methods for detecting CTCs**

| System | Capture efficiency | Autonomous movement | Clinical Sample Detection | Ref |
| --- | --- | --- | --- | --- |
| ICP-MS | 63.9% for MCF-7 (200,000-800,000 cells) | No | No | ^[1]^ |
| MNPA-TCMMGO | 78.0% for MCF-7 (5-200 cells) | No | No | ^[2]^ |
| SiO_2_NR@MB@APBA | 63.0-74.0% for MCF-7 (50-500 cells) | No | Yes | ^[3]^ |
| CIAH | 69.2% for MCF-7 (50-500 cells) | No | Yes | ^[4]^ |
| 3D Electrode | 71.0% for SKBR3 (10-500 cells) | No | Yes | ^[5]^ |
| ApTDN-Chip | 73.0% for SW480 (100 cells) | No | Yes | ^[6]^ |
| MMDA Hunter | 90.2-100.0% for SKBR3, 94.1-100.0% for HepG2, 95.1-100.0% for HT29 (10-5,000 cells) | Yes | Yes | This Work |

ICP-MS: inductively coupled plasma mass spectrometry. MNPA-TCMMGO: a multivalent nano-plate aptamer functionalized biomimetic magnetic graphene oxide platform. SiO_2_NR@MB@APBA: an aminophenylboronic acid functionalized SiO_2_ nanorods@microbowls microarray. CIAH: a cell-imprinted alginate hydrogel. 3D Electrode: a new form of channeled and conductive three-dimensional electrode. ApTDN-Chip: tetrahedral DNA nanostructure with a pendant aptamer at the top vertex, deterministic lateral displacement-patterned microfluidic chip.

**Table S2. Oligonucleotide sequences involved in the research**

| Name | Sequence (5'-3') |
| --- | --- |
| L3-L-Apt_EpCAM_ | AGGCACCATCGTAGGTTTCTTGCCAGGCACCATCGTAGGTTTCTTGCCAGGCACCATCGTAGGTTTCTTGCCAGTATGCGTTCACTACAGAGGTTGCGTCTGTCCCACGTTGTCATGGGGGGTTGGCCTG |
| L3 | AGGCACCATCGTAGGTTTCTTGCCAGGCACCATCGTAGGTTTCTTGCCAGGCACCATCGTAGGTTTCTTGCC |
| Biotin-N2 | Biotin-TTTAGCAACCTGCCTGGCAAGCCTACGATGGACACGGTAATGAC |
| N3A | GTCATTACCGTGTGGTTGCTAAAAAAAAAAA |
| N3T | GTCATTACCGTGTGGTTGCTATTTTTTTTTT |

**Table S3. Information of cancer patients**

| Number | Gender | Age  (year) | Type | Clinical stage | Histopathological diagnosis  (Yes/No) | Capture CTCs |
| --- | --- | --- | --- | --- | --- | --- |
| 1 | Female | 39 | Breast cancer | ⅡA | Yes | 1 |
| 2 | Male | 62 | Lung cancer | Ⅳ | Yes | 3 |
| 3 | Female | 58 | Lung cancer | ⅣB | Yes | 3 |
| 4 | Female | 63 | Breast cancer | ⅢB | Yes | 7 |
| 5 | Male | 72 | Rectal cancer | ⅢA | Yes | 5 |
| 6 | Male | 67 | Rectal cancer | Ⅳ | Yes | 3 |
| 7 | Male | 76 | Lung cancer | ⅢA | Yes | 2 |
| 8 | Female | 31 | Breast cancer | ⅡA | Yes | 1 |
| 9 | Female | 60 | Ovarian cancer | Ⅳ | Yes | 4 |
| 10 | Female | 51 | Colon cancer | Ⅳ | Yes | 4 |
| 11 | Male | 66 | Lung cancer | ⅡB | Yes | 1 |
| 12 | Male | 54 | Esophageal cancer | ⅢA | Yes | 1 |
| 13 | Male | 80 | Rectal cancer | ⅢB | Yes | 1 |
| 14 | Female | 71 | Rectal cancer | Ⅳ | Yes | 2 |
| 15 | Male | 40 | Oral cancer | I | Yes | 2 |
| 16 | Male | 94 | Lung cancer | ⅢA | Yes | 4 |
| 17 | Male | 51 | Gastric cancer | Ⅳ | Yes | 2 |
| 18 | Female | 73 | Breast cancer | Ⅳ | Yes | 4 |
| 19 | Female | 64 | Thyroid cancer | Ⅳ | Yes | 2 |
| 20 | Male | 67 | liver cancer | ⅢA | Yes | 1 |
| 21 | Female | 59 | Breast cancer | ⅢA | Yes | 3 |
| 22 | Male | 76 | Rectal cancer | ⅢA | Yes | 3 |
| 23 | Male | 66 | Rectal cancer | Ⅳ | Yes | 2 |
| 24 | Female | 41 | Breast cancer | ⅡB | Yes | 4 |
| 25 | Female | 69 | Breast cancer | ⅡA | Yes | 2 |
| 26 | Male | 58 | Lung cancer | ⅢB | Yes | 7 |
| 27 | Female | 42 | Breast cancer | Ⅰ | Yes | 2 |
| 28 | Female | 83 | Pancreatic cancer | ⅡA | Yes | 3 |
| 29 | Male | 67 | Gastric cancer | ⅢA | Yes | 1 |
| 30 | Male | 53 | Lung cancer | Ⅳ | Yes | 1 |
| 31 | Female | 69 | Lung cancer | ⅢB | Yes | 3 |
| 32 | Male | 58 | Lung cancer | Ⅳ | Yes | 2 |
| 33 | Female | 61 | liver cancer | ⅡA | Yes | 1 |
| 34 | Male | 67 | Lung cancer | ⅢA | Yes | 1 |
| 35 | Female | 42 | Breast cancer | ⅡA | Yes | 1 |
| 36 | Male | 46 | liver cancer | ⅡA | Yes | 2 |
| 37 | Male | 65 | Lung cancer | Ⅳ | Yes | 7 |
| 38 | Female | 59 | Breast cancer | ⅢB | Yes | 6 |
| 39 | Male | 65 | Bladder cancer | Ⅳ | Yes | 3 |
| 40 | Female | 58 | Breast cancer | ⅡA | Yes | 6 |
| 41 | Female | 46 | Breast cancer | ⅢB | Yes | 9 |

CTCs were captured using this work.

**Table S4. Information of healthy controls**

| Number | Gender | Age  (year) | Type | Hematology and imaging index  (Normal/Abnormal) | Capture CTCs |
| --- | --- | --- | --- | --- | --- |
| H1 | Male | 36 | Healthy donor | Normal | 0 |
| H2 | Male | 29 | Healthy donor | Normal | 0 |
| H3 | Female | 44 | Healthy donor | Normal | 0 |
| H4 | Female | 30 | Healthy donor | Normal | 0 |
| H5 | Female | 41 | Healthy donor | Normal | 0 |
| H6 | Female | 41 | Healthy donor | Normal | 0 |
| H7 | Female | 42 | Healthy donor | Normal | 0 |
| H8 | Male | 56 | Healthy donor | Normal | 0 |
| H9 | Female | 55 | Healthy donor | Normal | 0 |
| H10 | Male | 50 | Healthy donor | Normal | 0 |
| H11 | Male | 39 | Healthy donor | Normal | 0 |
| H12 | Male | 60 | Healthy donor | Normal | 0 |
| H13 | Female | 55 | Healthy donor | Normal | 0 |
| H14 | Male | 26 | Healthy donor | Normal | 0 |
| H15 | Male | 43 | Healthy donor | Normal | 0 |
| H16 | Male | 40 | Healthy donor | Normal | 0 |
| H17 | Male | 62 | Healthy donor | Normal | 0 |
| H18 | Male | 33 | Healthy donor | Normal | 0 |
| H19 | Female | 30 | Healthy donor | Normal | 0 |
| H20 | Female | 27 | Healthy donor | Normal | 0 |
| H21 | Female | 52 | Healthy donor | Normal | 0 |
| H22 | Male | 35 | Healthy donor | Normal | 0 |
| H23 | Female | 42 | Healthy donor | Normal | 0 |
| H24 | Male | 29 | Healthy donor | Normal | 0 |
| H25 | Male | 51 | Healthy donor | Normal | 0 |
| H26 | Female | 46 | Healthy donor | Normal | 0 |
| H27 | Female | 24 | Healthy donor | Normal | 0 |
| H28 | Male | 50 | Healthy donor | Normal | 0 |
| H29 | Male | 26 | Healthy donor | Normal | 0 |
| H30 | Male | 24 | Healthy donor | Normal | 0 |
| H31 | Male | 41 | Healthy donor | Normal | 0 |
| H32 | Male | 46 | Healthy donor | Normal | 0 |
| H33 | Male | 37 | Healthy donor | Normal | 0 |
| H34 | Male | 38 | Healthy donor | Normal | 0 |
| H35 | Female | 59 | Healthy donor | Normal | 0 |
| H36 | Male | 42 | Healthy donor | Normal | 0 |
| H37 | Male | 30 | Healthy donor | Normal | 0 |
| H38 | Male | 34 | Healthy donor | Normal | 0 |
| H39 | Female | 37 | Healthy donor | Normal | 0 |
| H40 | Female | 64 | Healthy donor | Normal | 0 |
| H41 | Female | 23 | Healthy donor | Normal | 0 |

CTCs were captured using this work.

**Reference:**

[1] X. Yin, B. Chen, M. He, B. Hu, *Anal. Chem.* **2020**, *92*, 10308.

[2] L. Jia, X. Zhen, L. Chen, Q. Feng, W. Yuan, Y. Bu, S. Wang, X. Xie, *J. Colloid Interface Sci.* **2023**, *631*, 55.

[3] R. Jin, J. Wang, M. Gao, X. Zhang, *Anal. Chem.* **2020**, *92*, 3403.

[4] S. Gao, S. Chen, Y. Liu, H. Mao, Q. Lu, *ACS Appl. Mater. Interfaces* **2021**, *13*, 19603.

[5] X. Wang, T. Gao, J. Zhu, S. Long, S. Zhao, L. Yuan, Z. Wang, *Anal. Chem.* **2023**, *95*, 2496.

[6] J. Zhang, B. Lin, L. Wu, M. Huang, X. Li, H. Zhang, J. Song, W. Wang, G. Zhao, Y. Song, C. Yang, *Angew. Chem. Int. Ed.* **2020**, *59*, 14115.
